# A Reappraisal of the Purported Gastric Pellet with Pterosaurian Bones from the Upper Triassic of Italy

**DOI:** 10.1371/journal.pone.0141275

**Published:** 2015-11-11

**Authors:** Borja Holgado, Fabio Marco Dalla Vecchia, Josep Fortuny, Federico Bernardini, Claudio Tuniz

**Affiliations:** 1 Mesozoic Research Group, Institut Català de Paleontologia 'Miquel Crusafont' (ICP), C/ Escola Industrial 23, E-08201 Sabadell, Catalonia, Spain; 2 Department of Geology, Universitat de València, C/ Doctor Moliner 50, E-46100 Burjassot, Valencia, Spain; 3 Centro Fermi, Museo Storico della Fisica e Centro di Studi e Ricerche “Enrico Fermi", Piazza del Viminale 1, 00184 Rome, Italy; 4 The ‘Abdus Salam’ International Centre for Theoretical Physics (ICTP), Multidisciplinary Laboratory, via Beirut 31, I-34014 Trieste, Italy; 5 Centre for Archaeological Science, School of Earth and Environmental Sciences, University of Wollongong, Wollongong, New South Wales 2522, Australia; New York Institute of Technology College of Osteopathic Medicine, UNITED STATES

## Abstract

A small accumulation of bones from the Norian (Upper Triassic) of the Seazza Brook Valley (Carnic Prealps, Northern Italy) was originally (1989) identified as a gastric pellet made of pterosaur skeletal elements. The specimen has been reported in literature as one of the very few cases of gastric ejecta containing pterosaur bones since then. However, the detailed analysis of the bones preserved in the pellet, their study by X-ray microCT, and the comparison with those of basal pterosaurs do not support a referral to the Pterosauria. Comparison with the osteology of a large sample of Middle-Late Triassic reptiles shows some affinity with the protorosaurians, mainly with *Langobardisaurus pandolfii* that was found in the same formation as the pellet. However, differences with this species suggest that the bones belong to a similar but distinct taxon. The interpretation as a gastric pellet is confirmed.

## Introduction

**Fig 1:**
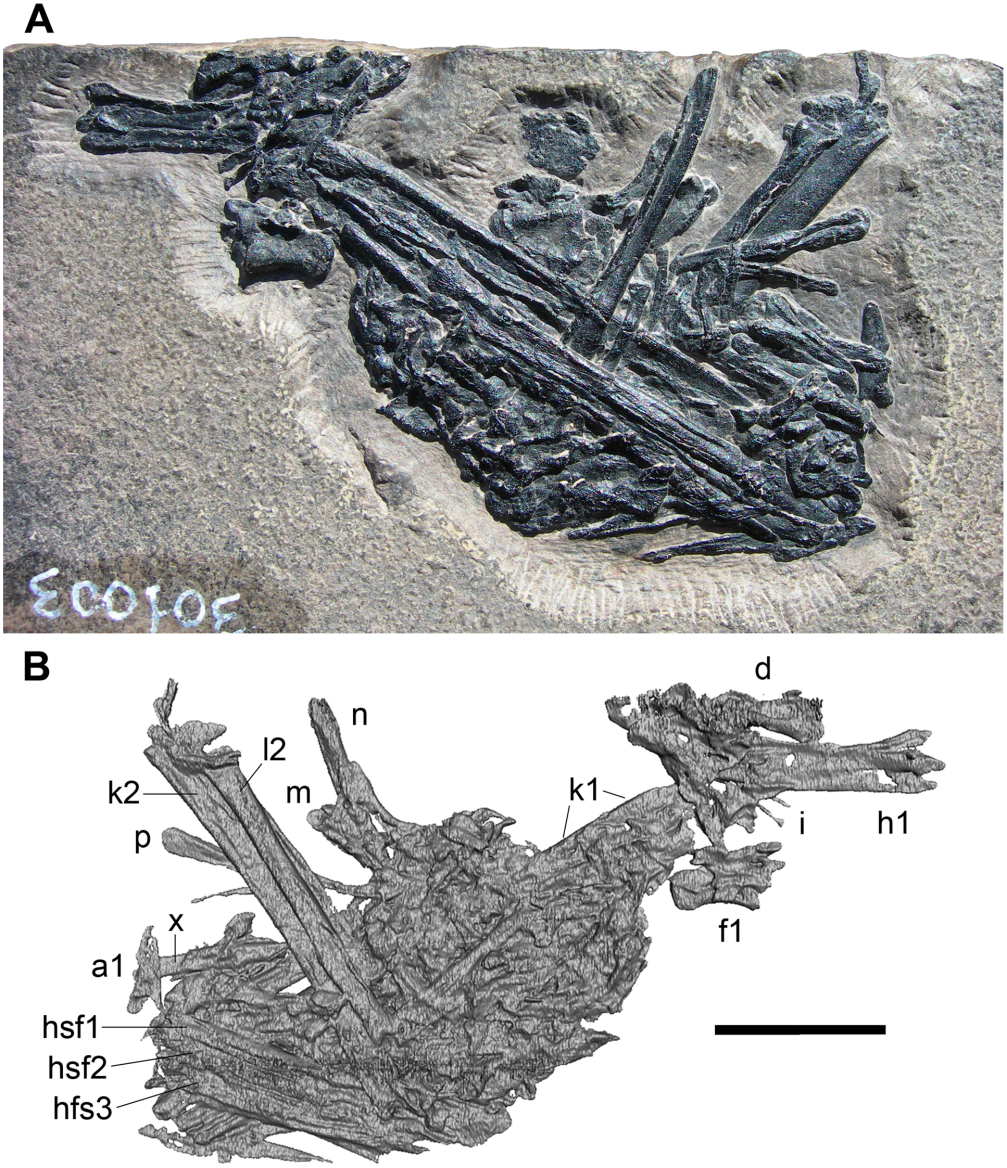
MFSN 1891, gastric pellet. Photo of the exposed surface (A), 3D rendering of the microCT virtual rendering of the other (hidden) surface (B). Abbreviations are explained in the text. The scale bar equals 10 mm.

In 1989, F.M. Dalla Vecchia, R. Wild and G. Muscio published [1] the description of a small pellet of bones (Fig 1A) from the Upper Triassic (Norian) Dolomia di Forni (Forni Dolostone) Formation of Friuli (NE Italy) as a gastric pellet made of pterosaur skeletal elements. They tentatively referred to it as cf. *Preondactylus buffarinii* Wild 1984 [2], the only pterosaur taxon known at the time from the Dolomia di Forni Formation. However, Dalla Vecchia [3] did not include it in his revision of *Preondactylus buffarinii*, and later [4] he referred it to an indeterminate pterosaur but did not describe the specimen further. The referral of the pellet to a pterosaur was accepted by many authors [5–13] who, however, had not seen the actual specimen and did not add further information about it. That fossil was mentioned as a rare case of gastric pellet containing pterosaurian bones in general textbooks and books on pterosaurs [14–17].

Dalla Vecchia [18] briefly reconsidered the specimen and compared it with the tetrapods found to that date in the Dolomia di Forni: the pterosaurs, the drepanosauromorph *Megalancosaurus preonensis*, and the protorosaurian *Langobardisaurus pandolfii*. His conclusions were that the referral to a pterosaur was still the most plausible because of the high number of long and slender bones from the limbs. In fact, a tetrapod usually has 12 long limb bones, while a pterosaur has 20 (see below). This statement was repeated by Dalla Vecchia [19–21]. However, he did not describe the specimen in more detail or revise the previous assignment of the skeletal elements. Dalla Vecchia [22] reported the pellet as pterosaur remains, but he underlined that a detailed study was in progress on the specimen.

Here we revise the former identification of each single skeletal element and try a referral based on an accurate description the skeletal elements and their comparison with those of a sample of Middle-Late Triassic reptiles. In order to do this, we also investigated the pellet by microCT analysis and subsequent manipulation of data.

Institutional abbreviations: BNM, Bündner Naturmuseum, Chur, Switzerland; BSP, Bayerische Staatssammlung für Paläontologie und Geologie, Munich, Germany; MCSNB, Museo Civico di Scienze Naturali, Bergamo, Italy; MFSN, Museo Friulano di Storia Naturale, Udine, Italy.

Anatomical abbreviations: be, broken extremity; cap, capitulum; ce, vertebral centrum; cf, caput femoris (head of the femur); con, condyle; cot, cotyle; cr, cervical rib; cv, cervical vertebra; de, distal end; di, diaphysis; dp, depression; dr, dorsal rib; dv, dorsal vertebra; fe, femur; fi, fibula; fo, foramen; g, gastralia; gr, groove; gtr, greater trochanter; h, humerus; hm, hemapophysis; ilb, indeterminate long bone; lb, laminar (sheet-like) bone; na, neural arch; ns, neural spine; pe, pedicel; poz, postzygapophysis; pre, proximal end; prz, prezygapophysis; rd, ridge; sp, spine of the hemapophysis; ti, tibia; tp, transverse process; tub, tuberculum; zyg, zygapophysis.

## Methods and Terminology

The studied specimen is stored at the Museo Friulano di Storia Naturale (MFSN) at Udine (Italy), under the catalog number MFSN 1891. The specimen first reported in 1989 [1]. No permits were required for the present study. The slab was analyzed by an X-ray microCT system at the Multidisciplinary Laboratory (MLAB) of the ‘Abdus Salam’ International Centre for Theoretical Physics (ICTP) in Trieste (Italy) [23]. The data acquisition was carried out with a source voltage of 110 kV, a current of 90 μA, an exposure time of 1.5 seconds and recording 1800 projections of the sample over 360°. MicroCT slices were reconstructed using the software DigiXCT in 16-bit format, at an isotropic voxel size of 20 μm. Ring artefacts correction was applied in order to improve the image quality.

Virtual preparation of the remains, performed at the Institut Català de Paleontologia ‘Miquel Crusafont’ (ICP; Catalonia, Spain), was done using the software Avizo^®^ v.7.1 (FEI-Visualization Sciences Group Inc.) to separate the microCT dataset into regions of interest (ROI) [24]. The skeletal elements of interest were isolated as 3D models through the process of segmentation [24] in order to see their hidden sides (Fig 1B) and to check whether they continue inside the pellet or not.

In order to evaluate the reliability of the 3D rendering models, those of us working with the microCT data (BH and JF) did not see the actual specimen before completing the 3D renderings. The degree of match between the 3D rendering and the actual morphology of the exposed part of the bones (unambiguously observable under the binocular microscope) is indicative of the reliability of the 3D rendering of the hidden parts of the same bone.

During the process of segmentation, the separation of the bone from the matrix was sometimes problematic. Some areas where bone is very thin appeared as hollows in the 3D rendering. Another problem was that the separation or distinction of the skeletal elements was very difficult because they are fragmentary, closely packed in the pellet, and without intervening matrix. This is not a limitation of microCT scanning technique but it is particular to this specimen. Also, microCT scan resolution did not to allow observation of details of the small bones in cross-section; anyway, this observation would be hampered by their crushing, collapse and the consequent closure of the lumina and nearly fusion of the walls of the skeletal elements. Despite these problems, the 3D rendering of the hidden side of the pellet is well resolved (Fig 1B).

**Fig 2:**
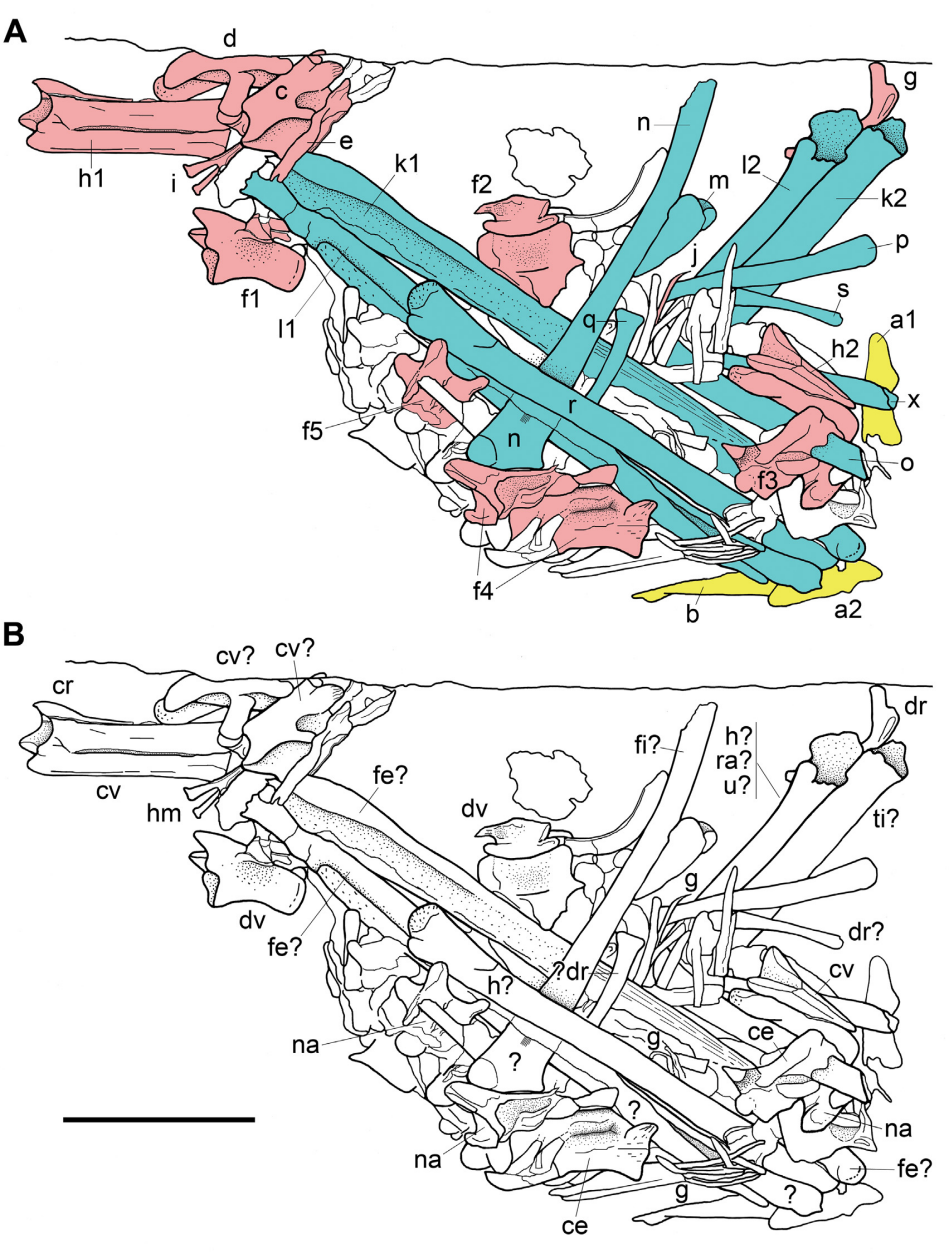
Interpretative drawings of MFSN 1891, gastric pellet. The pellet with the elements as interpreted by Dalla Vecchia et al. [1] and analyzed in the text (A); the elements according our interpretation (B). In A, purported cranial elements are in yellow colour, axial elements are in pink, and appendicular elements in blue; letters mark the elements as they were identified by Dalla Vecchia et al. [1] following the order of description in the text: palatines (a1, a2), pterygoid (b), cervical vertebrae (c and d), cervical rib (e), dorsal vertebrae (f1-f5), dorsal rib (g), caudal vertebrae (h1-h2), hemapophysis (i), gastralia (j), ulna (k1-k2), radio (l1-l2), wing metacarpal (m), wing phalanx 1 (n), wing phalanx 2 (o), wing phalanx 3 (p), wing phalanx 4 (q), femur (r), metapodial (s). A further element, which was not mentioned by Dalla Vecchia et al. [1], is marked with x. The drawing is based on Figure 3 of Dalla Vecchia et al. [1], redrawn and modified. Anatomical abbreviations are explained in the text. The scale bar equals 10 mm.

The skeletal elements exposed on the surface are described and discussed according to an anatomical order (first skull, then axial, and finally appendicular elements; forelimb elements before hind limb elements; limb elements are described according to their position from proximal to distal) following the identification as pterosaurian bones according to [1], notwithstanding it is potentially or actually wrong. The reasons of the referral of each element to a pterosaurian skeletal element by Dalla Vecchia et al. [1] are reported first; then, the element is described in detail, discussing the resemblance of bones or structures to the bones of pterosaurs or other taxa, if the case. In order to avoid confusion in the description, the elements were indicated with lower case letters of the alphabet (from a to s; see Fig 2A). Subsequently, each element is compared with the bones of basal pterosaurs to which it was referred by Dalla Vecchia et al. [1] in order to establish whether the referral is reliable or not. Finally, alternatives to the original attribution [1] are reported, if they are possible. The sample of Middle-Late Triassic reptiles utilized for comparison includes some lepidosauromorphs (kuehneosaurid and sphenodontians), thalattosaurians (*Endennasaurus*), *Pachystropheus*, trilophosaurs, rhynchosaurs, *Longisquama*, *Mecistotrachelos*, *Sharovipteryx*, drepanosauromorphs, protorosaurians, basal archosauriforms, phytosaurs, ornithosuchids, aetosaurians, poposauroids, rauisuchids, basal crocodylomorpha, *Scleromochlus*, early pterosaurs, basal dinosauromorphs, basal dinosauriformes, and basal dinosaurs.

The distribution of the bones in space (right, left, upper, lower etc.) is described according to their position in Fig 1A.

## Analysis of the Specimen and Comparison

The skeletal elements were not actually described by Dalla Vecchia et al. [1], who just tried to match them with those of non-pterodactyloid pterosaurs, in particular *Preondactylus buffarini* and the other Triassic taxa known in 1988 when the paper was written (i.e., *Eudimorphodon ranzii* and *Peteinosaurus zambellii*).

The elements of the pellet identified by Dalla Vecchia et al. [1] are listed here according such identification, they are described, and then compared to those of pterosaurs in order to check whether the original referral [1] is plausible or not. If a resemblance to elements of other reptiles is apparent, it is also mentioned.

### General description

The gastric pellet is lens-like, 52 mm long, 33 mm wide, and 3.2 mm of maximum thickness. The distribution of the bones within the pellet is chaotic, although there are preferential orientations for long bone remains. The identifiable elements are mainly partial vertebrae and portions of long bone shaft that are clumped in a background of smaller bone fragments (Figs 1 and 2). As it was observed by Dalla Vecchia et al. [1], there is no evidence of a background of amorphous organic matter surrounding the bones.

### Skull elements

**Palatines** (a1 and a2 in Fig 2A)

**Fig 3:**
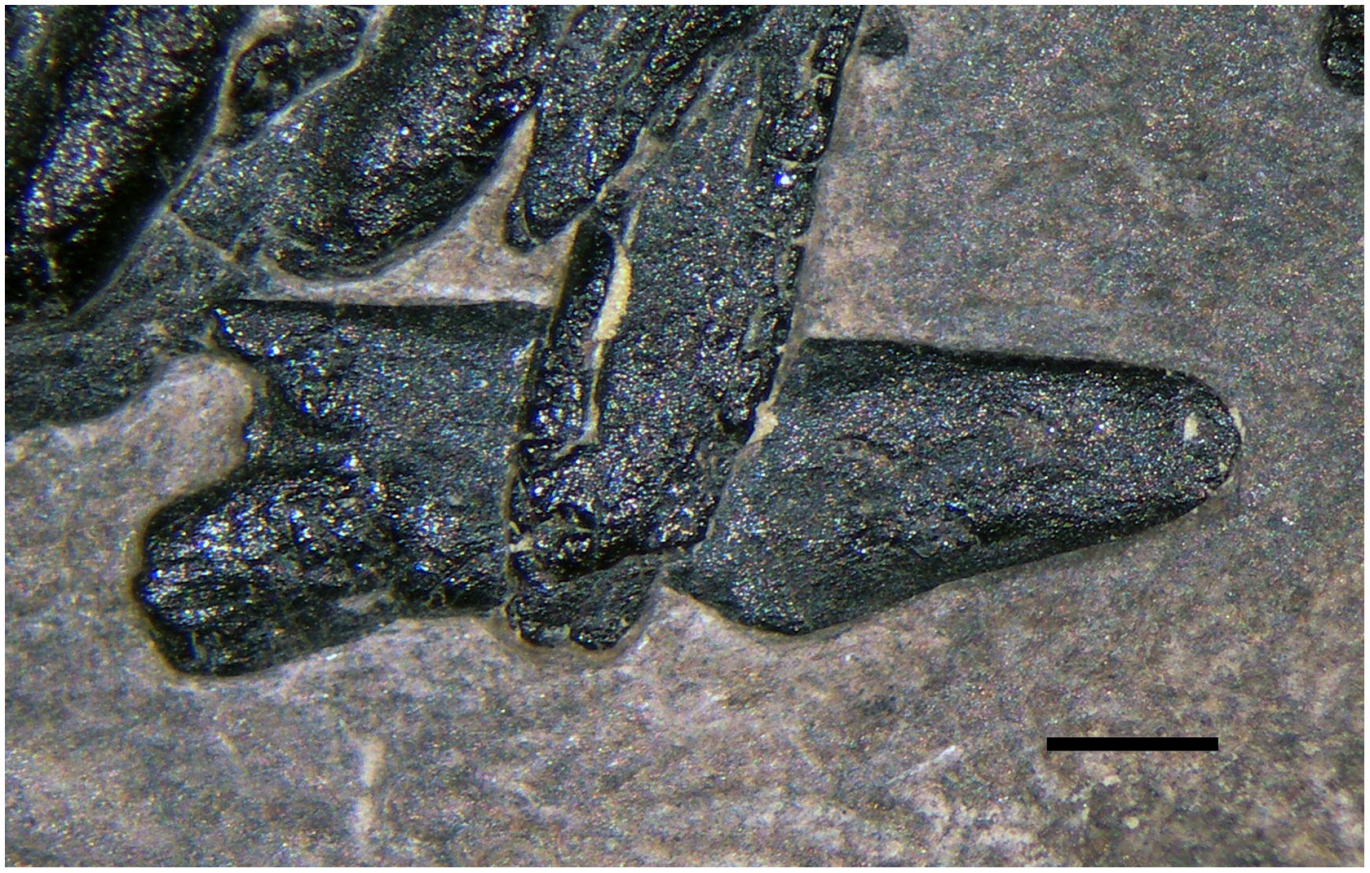
The purported palatine (a1 in Fig 2A). Photograph of the element. The scale bar equals 1 mm.

Two small bones of grossly similar morphology placed along the margin of the right lower corner of the pellet are identified as palatines in Figure 3 of Dalla Vecchia et al. [1], but they are not mentioned in the text. Their margins are smooth, thus they are plausibly not affected by breakage, i.e. they are not just misshapen bone fragments. The nearly completely exposed one (Fig 3) is 6.5 mm long, its maximum width is 2.2 mm and its maximum thickness is 0.5 mm. It is roughly triangular, narrow and elongated, with a blunt apical extremity; there is probably a notch at its wider extremity (left in Fig 3).

Palatines are not well-exposed in any Triassic pterosaurs [22]. Ősi et al. [24] described the palate of the non-pterodactyloid pterosaurs based on a three-dimensionally preserved specimen of the Early Jurassic *Dorygnathus banthensis*. The palatine of the latter ([25]: Figure 7) is unlike the two elements in MFSN 1891, whose identity remains unknown.

**Pterygoid** (b in Fig 2A)

As for the purported palatines, a small bone is identified as a pterygoid in Figure 3 of Dalla Vecchia et al. [1], but it is not mentioned in the text. It is a thin, slender, and pointed fragment of bone cropping out from the lower right corner of the pellet. The exposed portion is 8 mm long.

**Fig 4:**
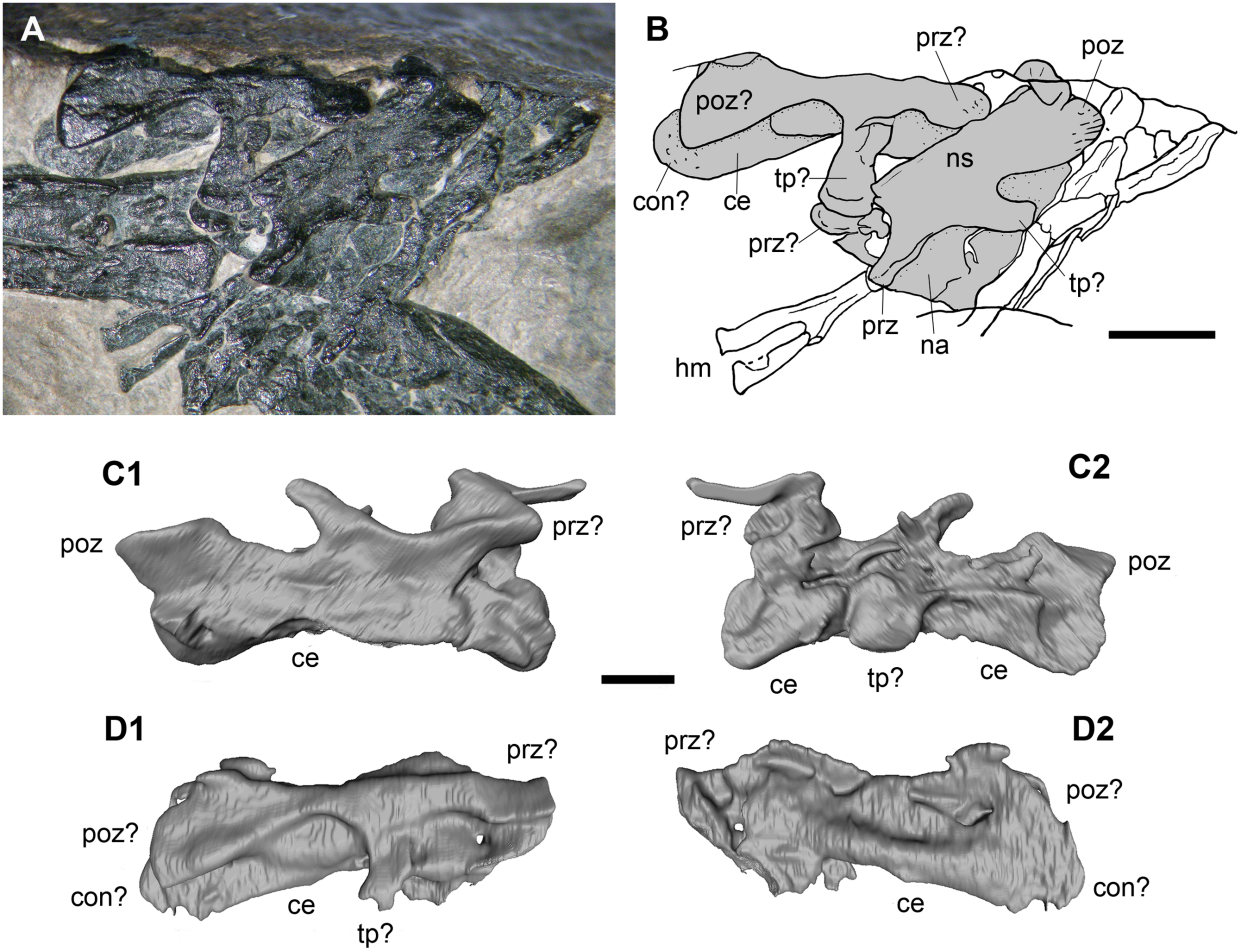
The purported cervical vertebrae (d and c in Fig 2A). The photograph (A); the interpretative drawing (B); the 3D rendering of the microCT dataset of c in the exposed (C1) and hidden (C2) views; the 3D rendering of the microCT dataset of d in the exposed (D1) and hidden (D2) views. In (B), the elements c and d are marked with the gray colour. Abbreviations are explained in the text. The scale bar equals 5 mm (B) and 1 mm (C-D).

No unequivocal features allow a referral to a pterosaurian pterygoid, other than the fact of being slender and pointed (see [26]: Figure 3; [27]: Figures 4 and 8). It is probably a fragment of bone belonging to the group of broken shafts that are located on the inner surface of the pellet (Fig 1B, hsf1-3), which crops out of the accumulation just near the purported pterygoid in the exposed view.

### Axial Skeleton

**Cervical vertebrae** (c and d in Fig 2A)

The two elements referred as cervical vertebrae in Figure 3 of Dalla Vecchia et al. [1] and in the text (page 123) are only partly preserved; they are strongly crushed, and probably broken. They occur close to each other and to the purported caudal vertebra (h1), which is better preserved and exposed. Their morphology is quite difficult to interpret.

The apparently most complete element (c in Fig 2A) resembles a relatively low neural arch in left lateral view with possibly the dorsal portion of the centrum (Fig 4A and 4B). It is 7 mm long from the prezygapophysis to the postzygapophysis, with a rectangular, long and low neural spine. The left prezygapophysis and the postzygapophyses are small, rounded, scarcely projecting, and with dorsally (prezygapophysis) and ventrolaterally (postzygapophyses) facing articular facets. The right prezygapophysis seems also be present, but it cannot be rejected that it is a zygapophysis from another vertebra covered by the surrounding elements, or even a broken and isolated zygapophysis. A longitudinal ridge connects the left prezygapophysis and postzygapophysis; a narrow and relatively elongate process (which is flattened against the neural arch) projects laterocaudally and ventrally from this ridge about at mid-arch. A similar process is shown on the other side by the 3D rendering (Fig 4C2). The segmented 3D rendering shows an elongated, tubular and arched structure in what should be the pedicel of the neural arch (Fig 4C). Apparently, it seems to be a long and low centrum with slightly expanded extremities, and possibly concave articular surfaces (so, the vertebra would be amphicoelous). However, it is evident from the direct observation of the exposed part of the element that this segmented 3D rendering is wrong in some details (e.g., the neural spine of element c was not detected; compare Fig 4A, 4B and 4C1). This suggests that the interpretations based on the 3D rendering are sometimes unreliable. In particular, very thin bone often cannot be distinguished from the rock.

The exposed portion of the other element (d) is quite enigmatic, appearing as triradiate (Fig 4A and 4B). Its total length is 8 mm. It is somewhat similar to the neural arch of element c, with the left prezygapophysis that is twisted laterally because of crushing. Actually, that zygapophysis could be a twisted right postzygapophysis, as suggested by further comparison (see below). The neural spine is missing. The narrow and long process that projects laterally (now ventrally because of crushing) from the longitudinal ridge is comparatively longer and more robust than that of the element c. The segmented 3D rendering shows that the underlying bone is also part of the element d. It appears as a tubular structure with a convex left extremity, which is plausibly the centrum of the vertebra. If this centrum is to be considered opisthocoelous or procoelous depends upon the identification of the zygapophyses. Comparison with the vertebra labelled h2 in Fig 2A (where the convex extremity of the centrum is the caudal one), suggests it is a procoelous vertebra.

The cervical vertebrae of the Triassic pterosaurs have a peculiar morphology ([22]: Figures 4.18A-B, 28, 37, 85, 106, 141 and 162A-C; [28]: Figure 6), which is also found in Early Jurassic taxa [27, 29–30]. In ventral view, their procoelous centrum is expanded cranially, constricted in the middle and expanded again at its rounded distal condyle. In dorsoventral view, the neural arch is stout and quadrangular and bears robust zygapophyses reminiscent of the buffers of a train that have subvertical articular facets. The neural spine is low with respect to the size of the vertebra, and the diapophysis and parapophysis are barely distinguishable. Those cervical vertebrae appear quadrangular also in lateral view ([22]: Figures 4.1.137 and 41) and are much larger than the dorsal vertebrae. The purported cervical vertebrae of MFSN 1891 show no features of the cervical vertebrae of a Triassic pterosaur. They appear to be relatively low and elongated vertebrae as the purported caudals of Dalla Vecchia et al. [1] (h1 and h2 in Fig 2A; see below), but they possibly are from a more proximal position.

**Cervical rib** (e in Fig 2A)

This element is mentioned only in Fig 3 of Dalla Vecchia et al. [1]. It is an elongate group (7 mm long) of strap-like and overlapping small bones placed close to the purported cervical vertebrae (elements c and d). It was probably referred to a cervical rib by Dalla Vecchia et al. [1] just because it is close to the purported cervical vertebrae. It is apparently made of a folded lamina of bone and it is not definitely filiform, or a bundle of filiform structures. The cervical ribs of Triassic pterosaurs have long filiform shafts that form bundles along the ventrolateral sides of the cervical segment of the vertebral column [22]. Those filiform shafts cannot be recognized in the purported cervical rib/s of MFSN 1891.

**Dorsal vertebrae** (f1-f5 in Fig 2A)

Five elements were identified as dorsal vertebrae by Dalla Vecchia et al. ([1]: Figure 3), presumably based on their morphology. They are located along the marginal part of the pellet. The better preserved and exposed is the one labeled f1 in Fig 2A; the others will be described in clockwise order moving around the pellet.

**Fig 5:**
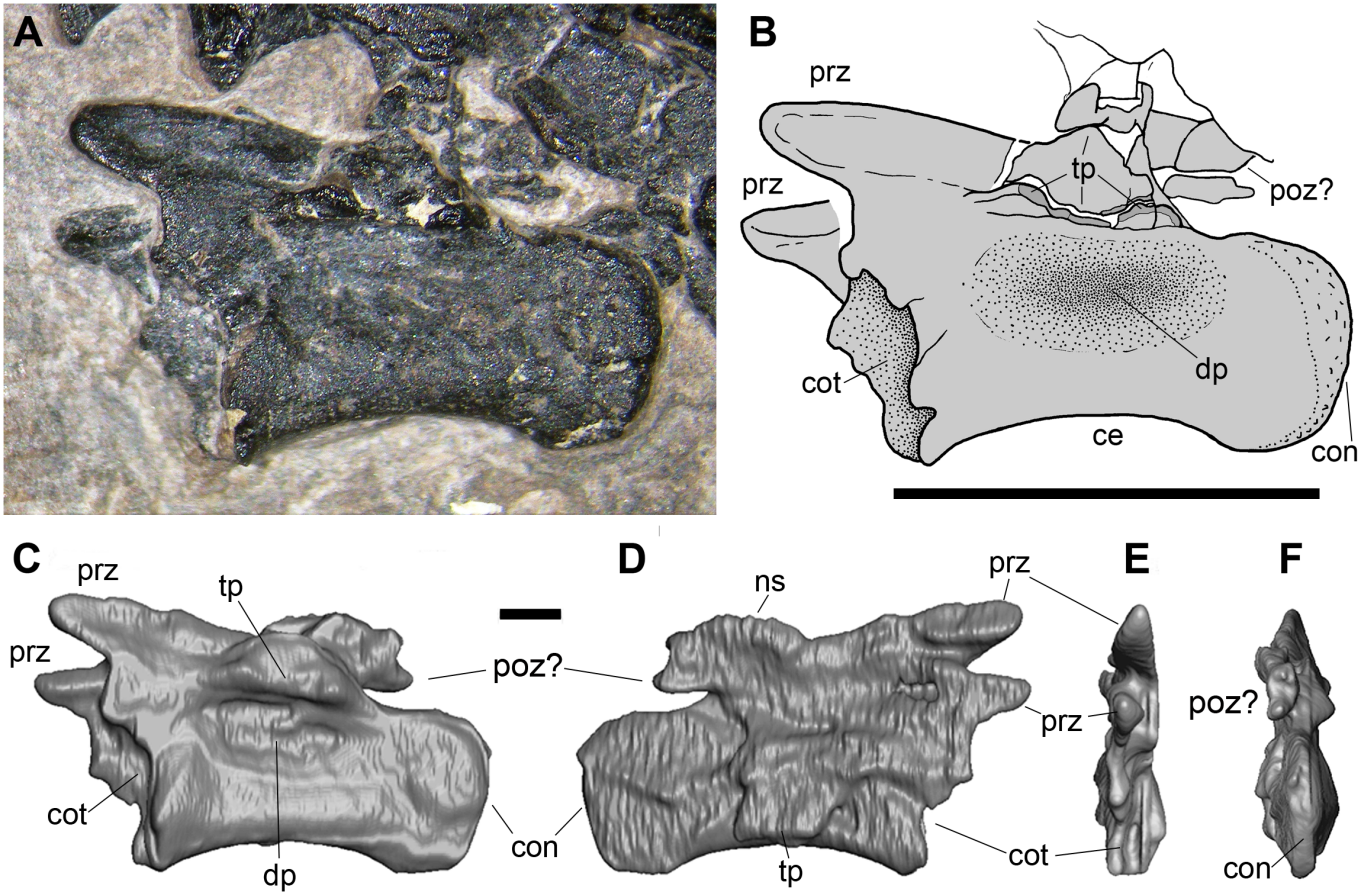
The better preserved dorsal vertebra (f1 in Fig 2A). Photograph of the specimen (A); interpretative drawing (B); the 3D rendering of the microCT dataset in left lateral view (exposed surface) (C), right lateral view (hidden surface) (D), cranial (E) and caudal (F) views. Abbreviations are explained in the text. The scale bar equals 5 mm (B) and 1 mm (C-F).

Element f1 is a procoelous vertebra exposed in left lateroventral view (Fig 5A and 5B). It is 7 mm long from the cranial extremity of the prezygapophyses to the caudal end of the centrum. The centrum is elongate, expanded at both extremities and constricted in the middle. It is 5.1 mm long and 2.5 mm high in the middle. The proximal extremity is deeper than the other and is clearly concave, while the distal one is convex and rounded. They are the cotyle and the condyle, respectively, in a ball-and-socket articulation. There is an elliptical fossa (which is emphasized in the microCT virtual rendering of Fig 5C) on the centrum just below the centrum-neural arch boundary, but no pneumatic foramina. The neural arch extends on the anterior 2/3 of the centrum and traces of an obliterated neurocentral suture are identifiable. The pedicel extends cranially up to the cranial end of the centrum. The prezygapophyses project well beyond the cranial end of the centrum, are straight and with a blunt extremity; the articular surface faces dorsomedially. The prezygapophyses resemble those of the close vertebra h1. At the base of the left side of the neural arch, the proximal part of a dorsoventrally flattened transverse process is preserved; more distal portions of the broken process are preserved nearby. The segmented 3D rendering shows the right transverse process, which is quadrangular (possibly broken distally), flattened dorsoventrally and slightly inclined caudally. According to the segmented 3D rendering, the neural spine is broken at its base and the only identifiable postzygapophysis (apparently the left one) is small and with a ventrally facing articular facet.

**Fig 6:**
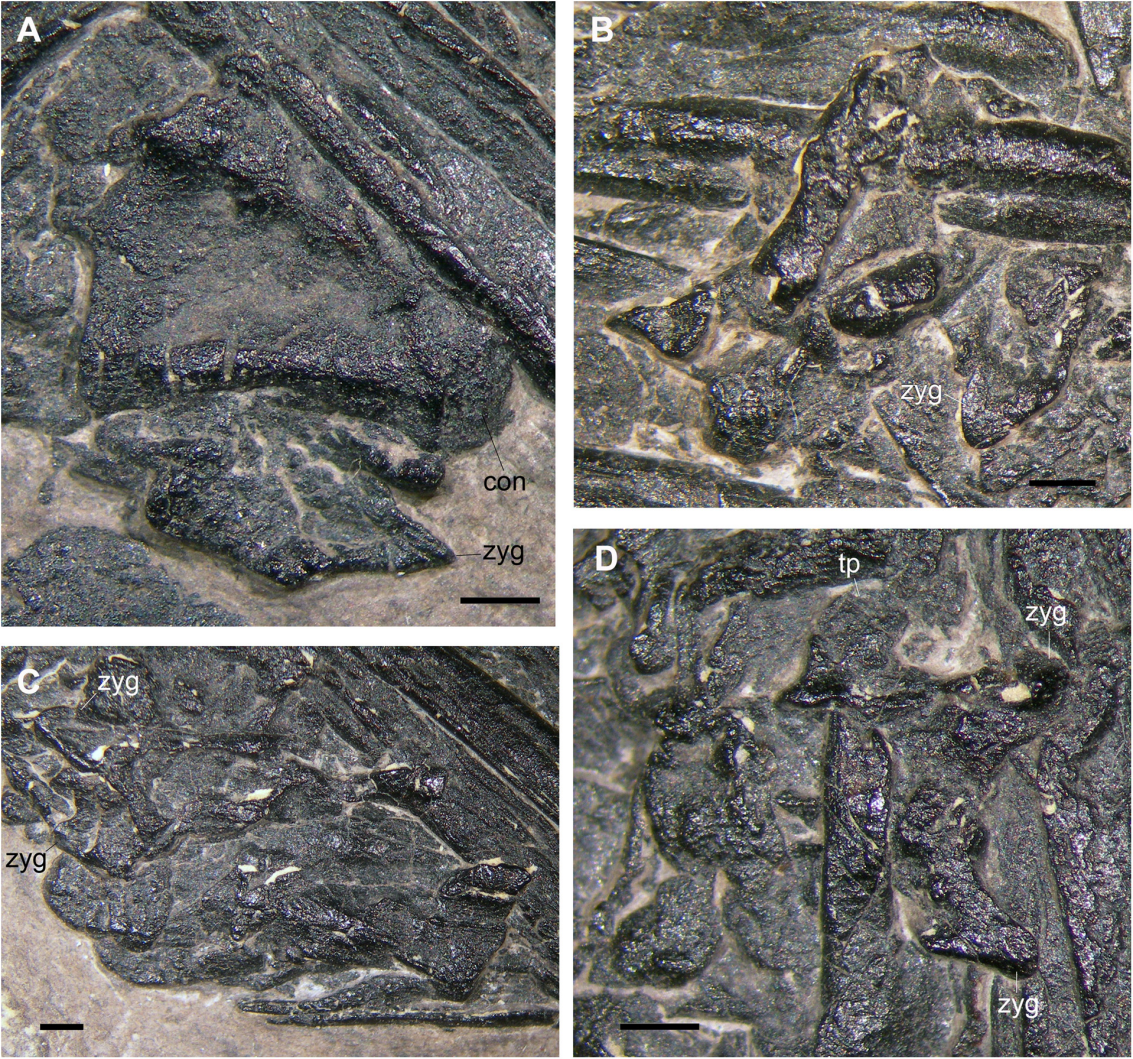
The other purported dorsal vertebrae (indicated as f2-f5 in Fig 2A). f2 (A); f3 (B); f4 (C); f5 (D). Abbreviations are explained in the text. The scale bar equals 1 mm.

The vertebra f2 is exposed in left lateral view (Fig 6A) and is similar to that just described. The centrum (5.5 mm long) has a concave ventral margin and is procoelous with an apparently hemispherical distal extremity. The pedicel of the neural arch seems to reach the cranial extremity of the centrum. At least the upper part of the left side of the neural arch seems to be broken. Part of a neural arch with a clearly recognizable right postzygapophysis comes out from the ventral margin of the centrum. It could be a broken portion of the neural arch of this vertebra or it could belong to a further vertebra laying below it, which cannot be distinguished in the microCT dataset, anyway.

The successive vertebra (f3) is poorly preserved and fragmentary (Fig 6B). It consists of a broken centrum, which is crushed against the long bones k1 and o, and portions of the arch. There is a tongue-like zygapophysis with a horizontal articular facet and possibly part of another zygapophysis.

The following vertebra (f4) is also a centrum crushed against other bones (Fig 6C). Dalla Vecchia et al. [1] did not notice a close partial neural arch in possible dorsal view with two paired zygapophyses similar to that present in element f3, possibly the postzygapophyses. It is unclear whether they belong to a further vertebra, whose centrum is mostly covered by the arch, or to the one indicated by Dalla Vecchia et al. [1].

The last vertebra (f5) is represented by a neural arch in dorsal view with a quadrangular outline (Fig 6D), similar to that just described above. The two postzygapophyses are short and rounded with a ventromedially facing articular facet. The right transverse process is tongue like, relatively short, and slightly curved caudally.

Only the first two allow comparison with the dorsal vertebrae of non-pterodactyloid pterosaurs. The others are too incompletely preserved and could be from other positions along the vertebral column. Vertebrae f1 and f2 are dorsals because of the position of the transverse process, absence of articular facets for the rib and hemapophysis in the centrum, their procoely, and the comparison with the other vertebrae preserved in the pellet, namely the more elongated ones. The dorsals of non-pterodactyloid pterosaurs are procoelous and resemble those described above, as far as it is possible to understand from the few available descriptions ([29]: Figure 2.19; [31]: Figure 6c-d; [32]: Figure 10). As there is no trace of the parapophysis on the centrum, they cannot be one of the first three dorsals of a pterosaur ([31]: Figure 6f). They differ from the other pterosaur dorsals in details: the centrum is more cylindrical in the latter; its distal extremity is less rounded or hemispherical; the pedicels are more centered on the neural arch and the cranial ones do not end at the very extremity of the centrum; the prezygapophyses are less projecting forward beyond the extremity of the centrum and the postzygapophyses are correspondingly placed at the level of the caudal end of the centrum; and the transverse process is located higher in the neural arch.

The pedicel extends cranially up to the cranial end of the centrum and connects the prezygapophysis to the extremity of the centrum also in the protorosaurian *Tanystropheus* ([33]: Figures 52–54; [34]: Figure 60), whose dorsals, however, are amphicoelous.

**Dorsal rib** (g in Fig 2A)

A dorsal rib is mentioned only in Figure 3 of Dalla Vecchia et al. [1]. It is a small bone mostly covered by the paired long bones k2-l2 in the upper right corner of the pellet. The proximal part appears to be that of a dicephalous rib with a distinct but short tuberculum and a long capitulum (see below). A short ridge runs along the proximodorsal margin of the shaft. The shaft is covered by the two long bones and crops out from l2, but the distal portion is not preserved. The shaft appears to be markedly arched. Its referral as a dorsal rib is plausible.

Triassic pterosaurs have dicephalous ribs with distinct but short tuberculum and a long capitulum and a somewhat curved shaft, at least in the mid-proximal dorsals ([32]: Plates 2, 8, 12 and 14, Figures 11a and 33). However, many other diapsids bear dicephalous dorsal ribs, including some protorosaurians, other basal archosauromorphs such as rhynchosaurs [35] and trilophosaurs [36], most basal archosauriforms (e.g., *Euparkeria* [37], *Proterochampsa* [38], ‘Proterosuchia’ [39], and *Vancleavea* [40]), most basal archosaurs [41–43], and dinosauromorphs [44–45].

**Caudal vertebrae** (h1 and h2 in Fig 2A)

Two elements were identified as caudal vertebrae by Dalla Vecchia et al. ([1]: Figure 3), who did not discuss the reasons for such identification.

**Fig 7:**
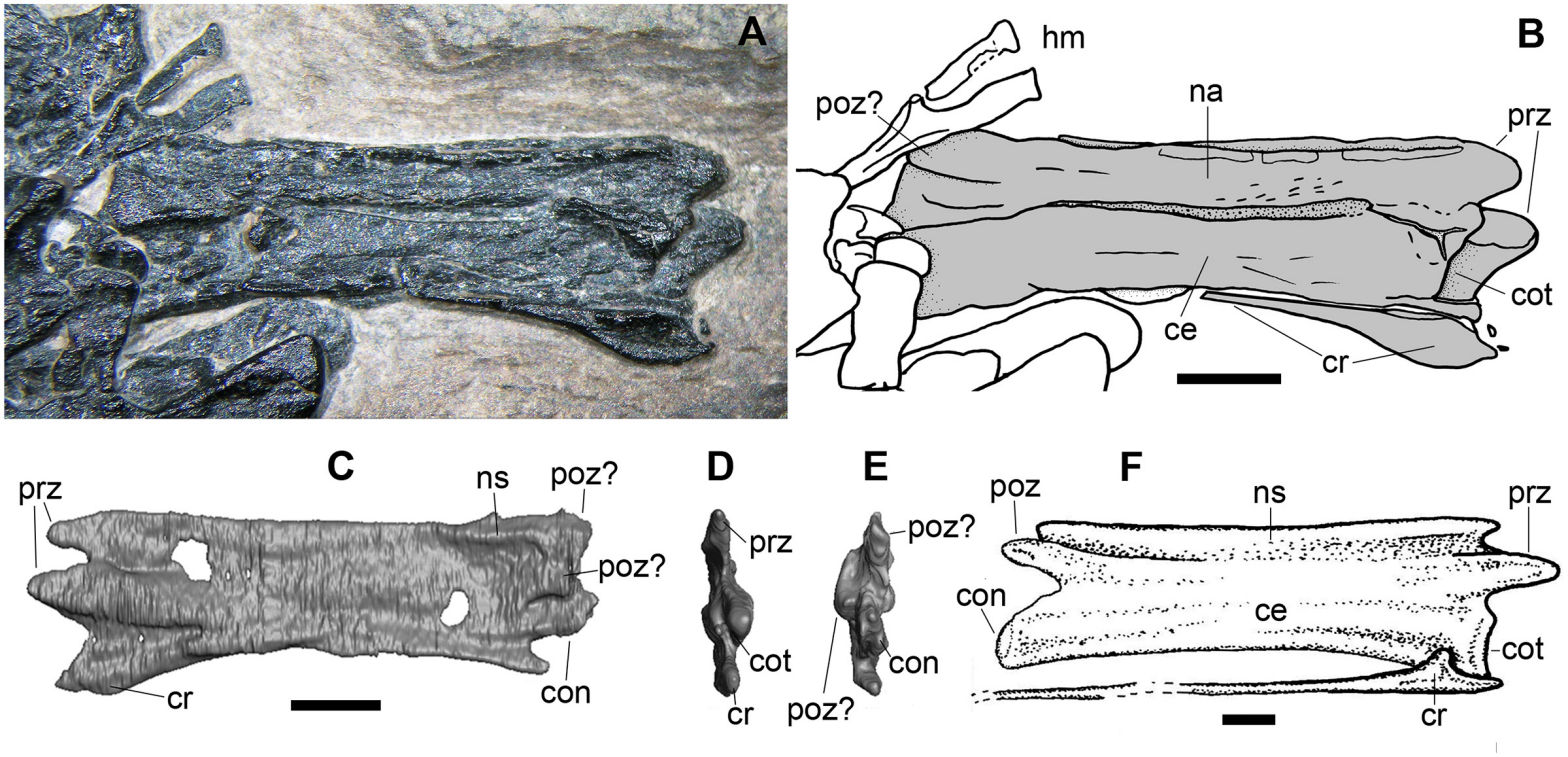
A purported caudal vertebra (h1 in Fig 2A). Photograph of the specimen (A); interpretative drawing (B); the 3D rendering of the microCT dataset in left lateral (hidden surface) (C), cranial (D), and caudal (E) views; 7th cervical vertebra of *Langobardisaurus pandolfii* in right lateral view and relative cervical rib (F). (F) is from Renesto [46]. Abbreviations are explained in the text. The scale bar equals 2 mm.

The vertebra here labeled h1 is nearly completely exposed, only one extremity being covered by other skeletal elements (Fig 7A and 7B). Its visible part is 11.8 mm long and its maximum height is 4 mm at the exposed extremity. It is a comparatively long and low bone that is compressed and crushed due to lithostatic pressure, as the many longitudinal fractures and its flatness suggest. Two paired and symmetrical processes project from the exposed extremity. An elliptical articular facet can be seen in one of them, suggesting that they are zygapophyses; the position of the process in the vertebra and the position of the facet in the process indicate that it is a left prezygapophysis with the articular facet facing mediodorsally, while the other process is the right prezygapophysis. This confirms the vertebral nature of the bone and shows its dorsoventral and craniocaudal polarity. The vertebra is exposed in right lateroventral view and appears to be upside-down in Figs 1 and 2. The remaining part of the element is composed mainly of the centrum and neural arch and is slightly expanded at the extremities. There is no neat distinction between centrum and neural arch, also because they are both flattened by crushing, but a groove seems to separate them longitudinally. The centrum was probably tubular-shaped and hollow; its total length is ~11 mm. Its cranial end appears to be concave, so it is procoelous or amphicoelous. Unfortunately, the caudal end of both centrum and neural arch are covered by other skeletal elements. In the microCT dataset, the articular surface of the caudal end of the centrum seems to be convex and small, but this is not very clear; the total length of the vertebra results to be 12.4 mm from the cranial extremity of the left prezygapophysis to the caudal end of the centrum. The neural arch is rectangular, low and strap-like, extending along the whole length of the centrum. The dorsal margin appears to be parallel to the ventral one. A short but thick longitudinal ridge occurs at the caudoventral margin of the neural arch (the caudal base of the pedicel). The neural spine cannot be distinguished on the exposed side because of the lateroventral exposition of the vertebra. According to the 3D rendering of the other side, it would be non-existent along most of the vertebral length, being possibly recognizable only just before the postzygapophyses (Fig 7C). The prezygapophyses point cranially and project beyond the cranial termination of the centrum. As anticipated above, the articular facets face mediodorsally. A triradiate fracture occurs just ventral to the right prezygapophysis, probably caused by the overlapping of a small bone fragment. According to the 3D rendering (Fig 7C–7E), the postzygapophyses are shorter and blunter than the prezygapophyses and they do not project beyond the centrum, but they could be broken. A hook-like process along the ventrocranial margin of the centrum resembles a cranially placed and well-developed hypapophysis like that present caudally in the cervicals of the drepanosauromorphs [46]. However, this structure is actually separated from the centrum by a narrow gap and shows an expanded cranial portion and a filiform posterior projection (Fig 7B and 7C). The expanded portion sends a small and pointed process cranially. Its overall morphology and position resembles that of the cervical ribs of tanystropheid protorosaurians (see Fig 7F; [33]: Figures 33, 35, and 101, Plates 4–5, 8 and 16–18; [34]: Figures 48 and 55; [47]: Figure 5).

**Fig 8:**
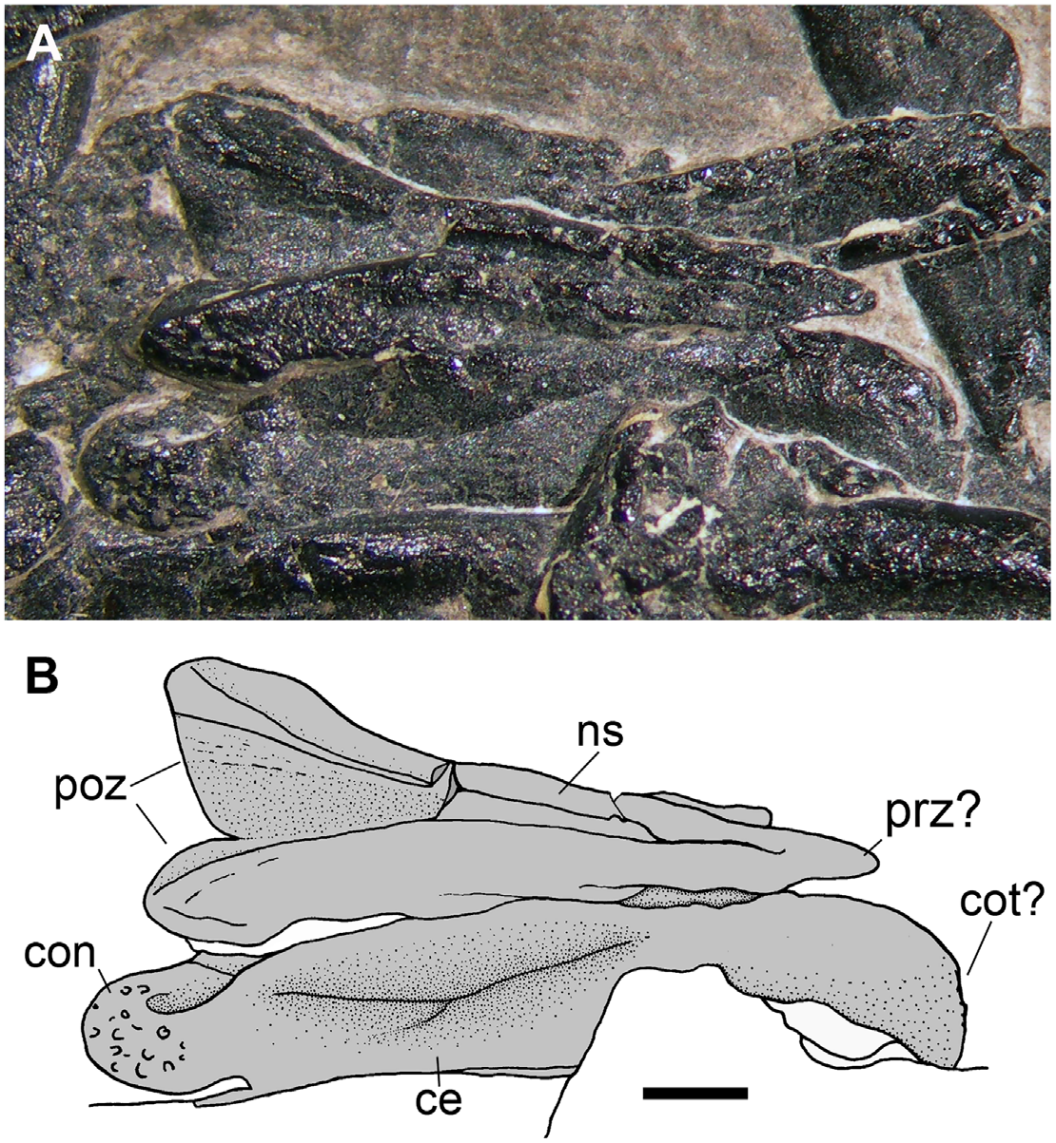
The other purported caudal vertebra (h2 in Fig 2A). Photograph of the specimen (A); interpretative drawing (B). Abbreviations are explained in the text. The scale bar equals 1 mm.

Only the neural arch in right dorsolateral view is clearly identifiable of the second putative caudal vertebra (h2; Fig 8A). The preserved part of this neural arch is 7 mm long. Two petaloid apophyses project from the arch, splayed laterally. They are postzygapophyses because there is no articular surface exposed, while there is a median dorsal ridge corresponding to the beginning of a spinopostzygapophysial lamina. Dorsally, the arch shows a longitudinal ridge that corresponds to a very low neural spine or a neural spine broken at its base. A cranial projection could be the remnant of a right prezygapophysis. A tubular bone, which is 8.5 mm long and lies just below the arch, might correspond to the centrum, as previously interpreted [1]. The bone has collapsed in the middle, so it was hollow inside. It has a prominent ball-like left end with small pits on its surface, which projects beyond the postzygapophyses. The other extremity appears to be rounded, but it is not ball-like and lacks pits. So, if this is the centrum of the overlying neural arch, the vertebra would be procoelous. The neural arch is clearly separated from it by a fracture, but this could be related to the crushing and/or the bite of the bone.

The vertebrae h1 and h2 were originally interpreted as caudal vertebrae because the caudal vertebrae of the pterosaurs, with the exception of a few proximal elements, are tubular and very elongated, with low neural arches and practically no neural spines [48]. However, the caudal vertebrae of non-pterodactyloid pterosaurs do not have hypapophyses and their hemapophyses have a triangular body and long cranial and caudal processes [26, 48]. Furthermore, the zygapophyses of the caudal vertebrae of Triassic pterosaurs are smaller than those of the vertebrae under examination, they are not splayed laterally, and they are pointed, at least in some cases [22, 48].

The overall morphology of the element h1 is that of the cervicals of *Langobardisaurus pandolfii* (Fig 7F), but with an even more reduced neural spine, as is the case of the cervicals of *Tanystropheus* [33]; this is in agreement with the identification of the structure along its ventral part as a cervical rib, because tanystropheids have filiform cervical ribs with an expanded dicephalous head articulating on a lateroventral facing and elongated diapophysis and parapophysis and with a small cranial prong ([33]: Figure 33).

**Hemapophysis** (i in Fig 2A)

**Fig 9:**
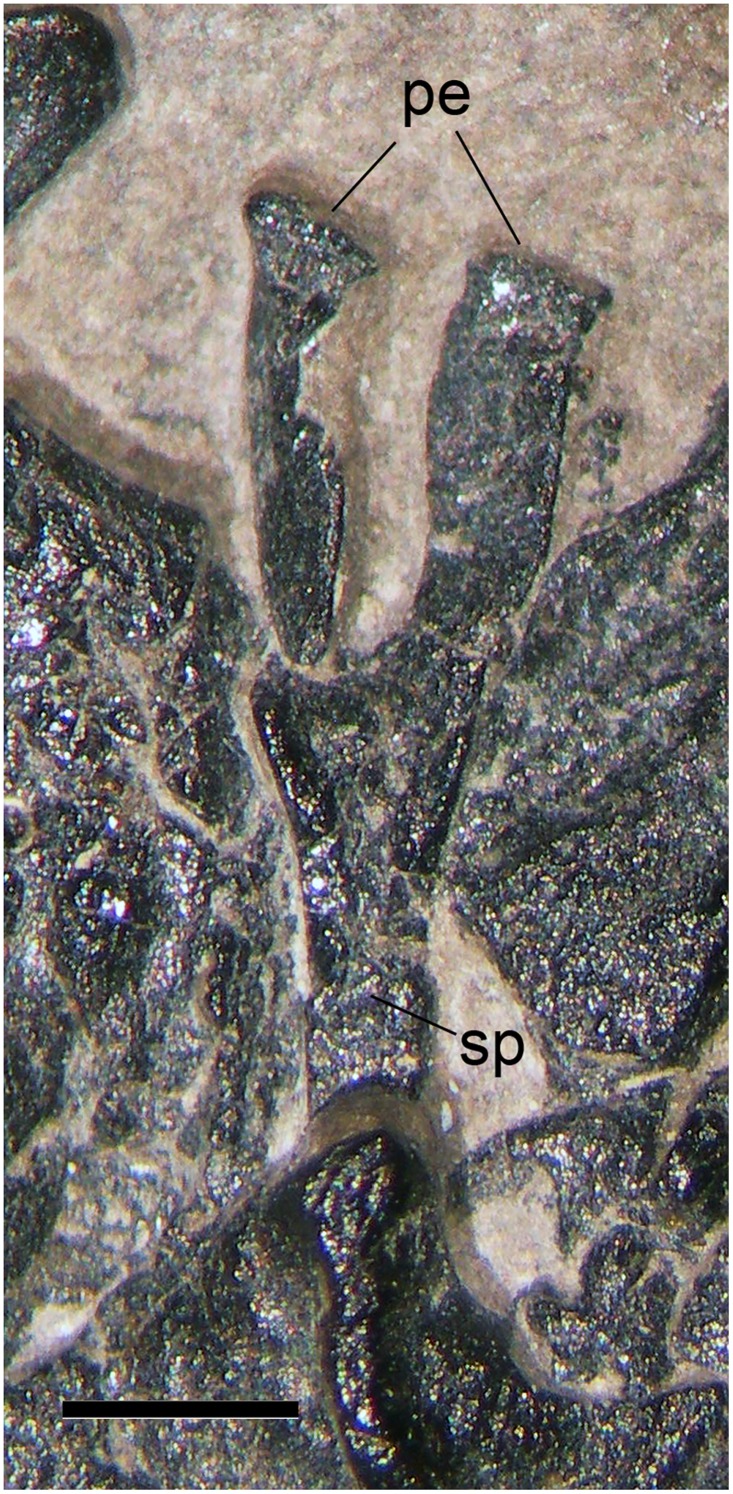
The hemapophysis (i in Fig 2A). Photograph of the specimen. Abbreviations are explained in the text. The scale bar equals 1 mm.

Dalla Vecchia et al. [1] mention this element only in Figure 3 of. It is a small, Y-shaped bone that is partially covered by element d (Figs 4A, 4B and 9). It has elongated and scarcely divergent pedicels (18°) that are expanded at the proximal extremity. The third branch of the Y is the spine, which is relatively long and narrow, both transversally and caudocranially; its covered distal end can be observed in the microCT virtual rendering or dataset. The total length of the bone is 8.5 mm; the spine is 4.9 mm long, but its unexpanded distal extremity seems to be broken, so it was probably longer. The identification of element i as a hemapophysis is evidently correct, having the plesiomorphic morphology for this element in reptiles [49].

Most of the hemapophyses of the pterosaur caudal vertebrae have a peculiar shape: a main body that is small and triangular in lateral view and long, filiform cranial and caudal processes on both left and right sides that are parallel to the vertebral centrum [31, 48]. Only those relative to the first caudals have a different morphology, but they are rarely well-preserved. One can be seen in the holotype of *Eudimorphodon ranzii* (MCSNB 2888; see [32]: Pl 2) between caudals 2 and 3. It is Y-shaped, but the pedicels are very short and the spine is quite stocky. The specimen MCSNB 3359 of *Peteinosaurus zambellii* has only one chevron without filiform processes, which is located between caudals 3 and 4 and is not Y-shaped (see [32]: Pl 14). No chevron can be identified in the proximal caudals of *Preondactylus buffarinii* (holotype, MFSN 1770; see [2]: Figure 3). The chevrons start from caudal 7 in *Rhamphorhynchus* and none is apparently Y-shaped [31]. No Y-shaped chevron is reported in *Dorygnathus* and *Campylognathoides* [27, 30]. The first hemapophyses of *Dorygnathus* are described as "triangular" by Padian, ([30]: page 53); they are absent in the reconstruction of Figures 22 and 23 [30]. The same occurs in the reconstruction of *Campylognathoides* ([27]: Figure 14). No Y-shaped proximal hemapophyses are reported in the skeletal reconstructions of basal pterosaurs in Witton [17].

At maximum, there would be one or two chevrons without filiform processes in a long-tailed pterosaur, so the chance of finding them in a pellet preserving only part of a skeleton would be low. On the other side, there are about 28 chevrons in the long tail of the protorosaur *Tanystropheus* at least half of which are similar to element i (see [33]: Plate 1, Figure 37).

**Gastralia** (j in Fig 2A)

Only one gastral element is identified in Figure 3 of Dalla Vecchia et al. [1] but they are more abundant, mainly in the right half of the pellet (Fig 2B). They are small and filiform bones, sometimes clearly ending with a sharp point. Pterosaurs have gastralia, as do many other diapsids [48].

### Appendicular skeleton

Several incompletely preserved long bones were referred to pterosaurian limb elements by Dalla Vecchia et al. [1].

**Radius-Ulna** (k1-l1 and k2-l2 in Fig 2A)

Two pairs of bones have been identified by Dalla Vecchia et al. ([1]: pages 123–125, Figure 3) as radius-ulna pairs, probably because they are composed by relatively slender and elongate bones, which are paired without a spatium interosseum, and one (the purported radius) seems to be slightly less robust than the other (the purported ulna), like the corresponding elements in pterosaurs [22, 32]. However, this is not explained in the text of the paper. Dalla Vecchia et al. ([1]: page 125) indicate the couple k1-l1 as the possible, nearly complete left radius and ulna.

**Fig 10:**
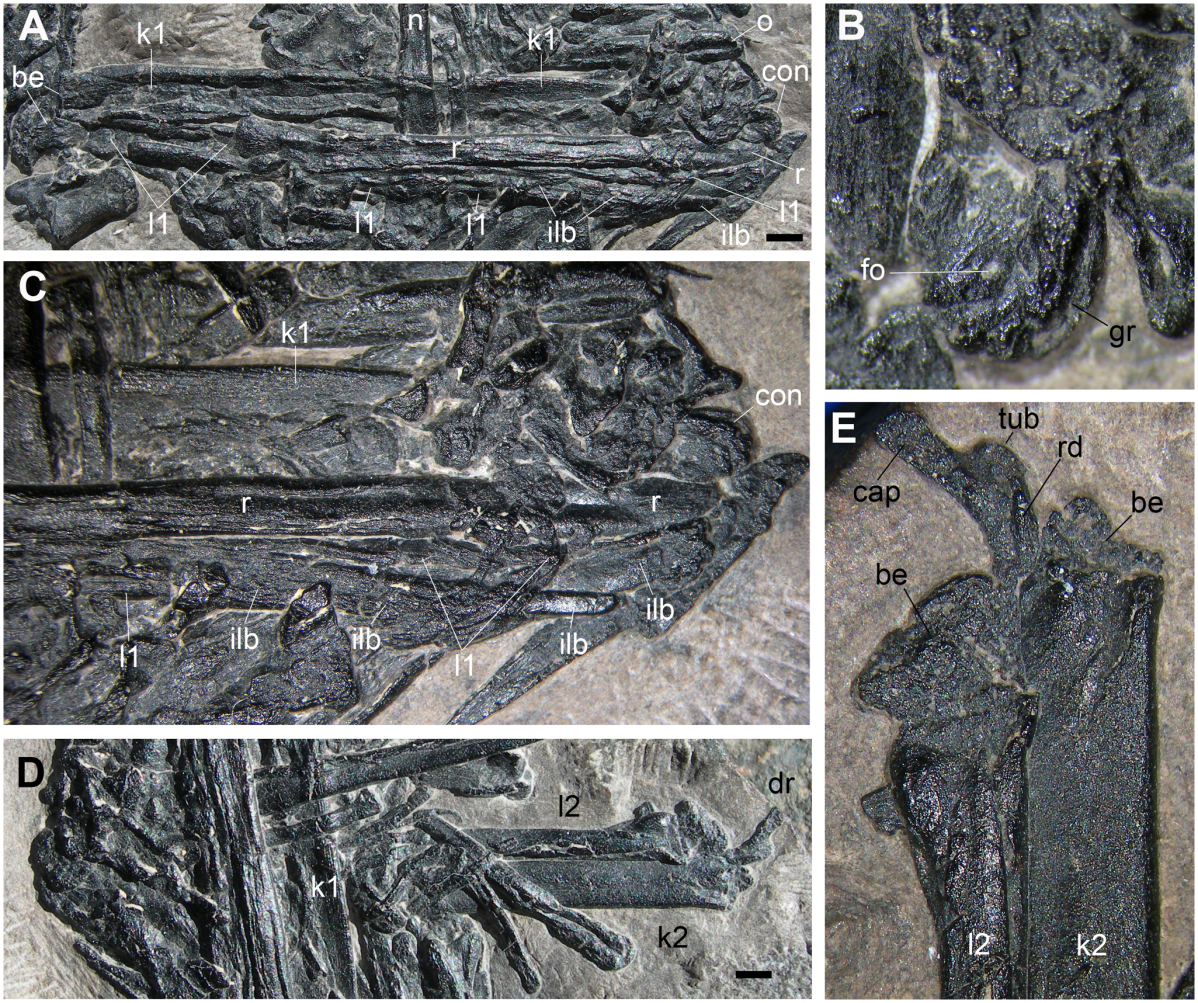
The purported radius-ulna pairs (l1-k1 and l2-k2 in Fig 2A). The pair l1-k1 (A); particular of the distal condyle of k1 (B); the distal portions of l1 and k1 and other elongate bone fragments parallel to them (C); the pair l2-k2 (D); the broken exposed extremities of l2 and k2, and the dorsal rib (g in Fig 2A) (E). Abbreviations are explained in the text. The scale bar equals 2 mm.

The couple k1-l1 crosses the pellet longitudinally. The practically complete k1 (Fig 10A) is 39.5 mm long according to the interpretation by Dalla Vecchia et al. [1], with a straight shaft that seems to arch slightly only distally where it ends with a condyle. The proximal part is slightly broader than the mid-shaft and is deeply collapsed in the middle; the proximal extremity ends against the purported cervical rib, and is probably broken. The surface of the mid-distal part of the shaft shows thin and long longitudinal ridges. The distal portion of the shaft is covered by other bones; according to Dalla Vecchia et al. [1], the distal extremity of the ulna (i.e., the condyle) seems to be in continuity with the shaft, but this cannot be ascertained, not even with the microCT data. The condyle (Fig 10B) is relatively small, rounded and slightly asymmetrically developed caudally; a groove seems to run along its cranial margin and a small foramen pierces its exposed side.

The purported radius (l1) lies alongside k1 proximally, but diverges slightly distally (Fig 10A). Its 'proximal' extremity is clearly broken and the 'proximal' segment of its shaft is totally collapsed, suggesting, as for the element k1, that it was hollow inside. Here, an elongate fragment of bone sticks out, which was referred to the 'radius' by Dalla Vecchia et al. [1], but it has blunt margins and a pitted surface and probably belongs to another element. Apart this 'proximal' segment contacting k1, the extent of the diaphysis of l1 is unclear, because there seems to be at least three segments of long bone shaft parallel to each other: the elements r and l1 and one indicated with the acronym ilb in Fig 10C; the latter overlaps l1 distally and tapers to a blunt extremity only 0.5 mm wide (Fig 10C). A further, distinct and long fragment crops out distally and is also marked with the abbreviation ilb in Fig 10C. The extremities of l1 appear to be broken; as preserved, the bone in about 36 mm long.

The exposed part of the couple l2-k2 is 17 mm long. The two elements are perfectly parallel to each other, k2 possibly overlapping slightly l2 (Fig 10C). The exposed extremities of k2 and l1 are broken (Fig 10D), as noticed also by Dalla Vecchia et al. [1]. The purported ulna (k2) is sensibly broader than the purported radius (l2); 8 mm from the exposed broken extremity of k2, the diameter of the latter is 2.5 mm, while that of l2 is 1.5 mm. The visible extremity of l2 is slightly expanded, while that of k2 is not. The diaphysis of the latter has a rather constant diameter throughout its length. The element k2 seems to be decidedly longer than l2 (29 vs. 23 mm) in the microCT virtual rendering (Fig 1B). The diaphysis of l2 does not seem to taper distally and appears to have a sort of bicondylar end in the microCT virtual rendering, while the 'distal' extremity of k2 could be broken (Fig 1B). The mid-'proximal' part of the diaphysis of l2 has a longitudinal groove along the side facing k2.

As we said above, those bones were identified as radius and ulna mainly because they are paired as the radius and ulna of pterosaurs. However, parallelism of long bones in a gastric pellet is a consequence of the constraints due to the morphology of the esophagus. As a consequence, long bones that were not in close anatomical relationships can result to be associated and parallel to each other. As noted above, l1 and k2 appears to be parallel to several other long bones (including the elements r and o). Also the elements n, q, l2, and k2 are parallel to each other. On the hidden surface, there seem to be a group of three or more parallel segments of long bone shafts gathered together (hsf1-3; Fig 1B), apparently all more or less the same size. When the ejecta fell down in the water and arrived on the sea bottom, it lost its compactness and original shape and partly broke up. Sets of closely bound and parallel long elements or fragments shifted and rotated with respect to each other, maintaining anyway the parallelism of the elements within each set.

As for size, l2 appears to be practically complete, but it is much shorter (23 mm) than the incomplete l1 (>36 mm). Therefore, they cannot belong to the same kind of skeletal element.

The radius and ulna of pterosaurs are easily identifiable based on the morphology of their extremities [22]. Nothing can be said in this sense for l1 and k2, as their extremities are damaged. The pterosaur radius does not have the fan-like (exposed) and apparently bicondylar (hidden) extremities of l2; the pterosaur ulna does not have the small distal condyle of k1.

All this does not support the identification of the elements l1-k1 and l2-k2 as paired radius-ulna of a pterosaur.

**Wing metacarpal** (m in Fig 2A)

**Fig 11:**
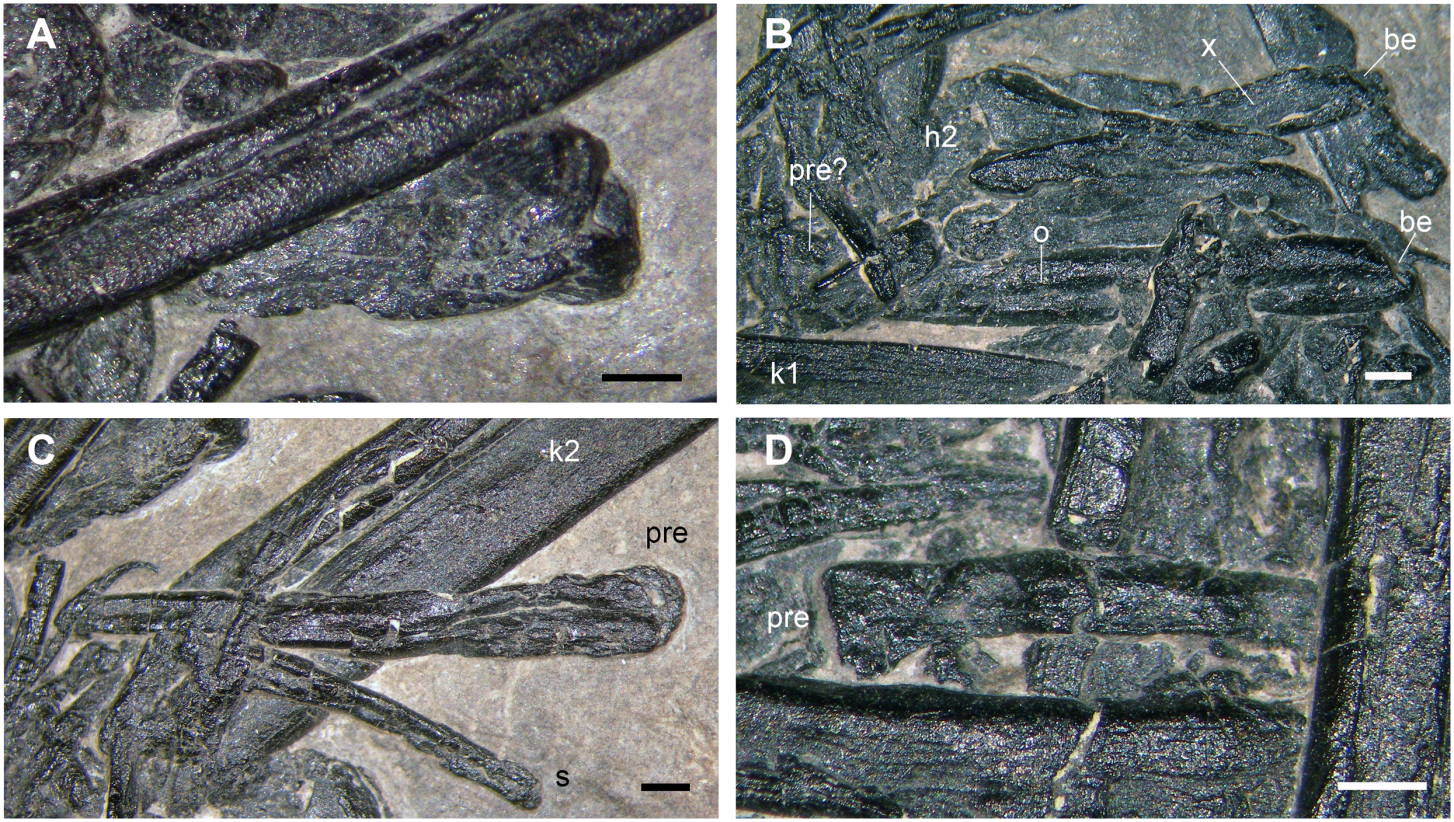
Other purported limb elements. The purported wing metacarpal (m in Fig 2A) (A); the purported wing phalanx 2 (o in Fig 2A and here), and the diaphysis fragment of another long bone (x in Fig 2A and here) (B); the purported wing phalanx 3 (p in Fig 2A) (C); the purported wing phalanx 4 (q in Fig 2A) (D). Abbreviations other than o and x are explained in the text. The scale bar equals 1 mm.

This element is mentioned only in Figure 3 of Dalla Vecchia et al. [1]. Element m was probably identified as a wing metacarpal because those authors recognized the characteristic distal condyles of that bone at the exposed extremity of the element. It is a relatively elongated, robust, and slightly arched bone (Fig 11A), which is partly covered by the element n. The exposed part is 5.8 mm long, while the total length is 6.3 mm according to the segmented 3D rendering. Its visible extremity appears to be divided into three parts surrounding a depression. No one of these parts can be reliably identified as a condyle. The wing metacarpals of Triassic pterosaurs have been recently described [28]: no features of this characteristic pterosaurian bone can be identified in the element m of the pellet. The extremity of the element is probably broken as a consequence of the bite of the predator/scavenger (see below).

**Wing phalanx 1** (n in Fig 2A)

**Fig 12:**
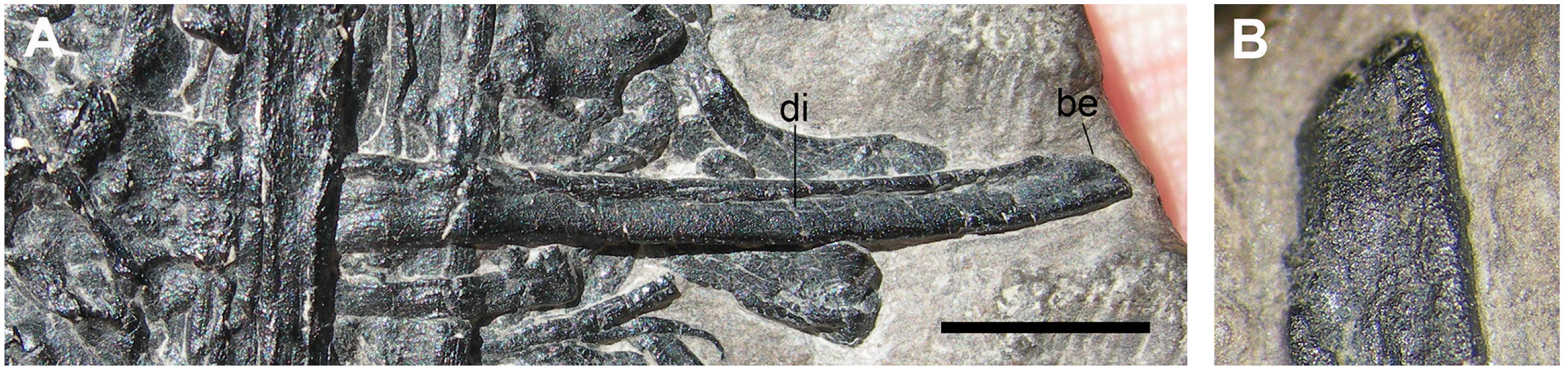
The purported wing phalanx 1 (n in Fig 2A). The whole element according to the interpretation of Dalla Vecchia et al. [1] (A); particular of the broken distal extremity (B). Abbreviations are explained in the text. The scale bar equals 5 mm.

This bone (Fig 12A) was identified as a wing phalanx 1 lacking its distal end ([1]: pages 125–126, Figure 3). Also in this case, the identification was based on its estimated length, which is between 28–30 mm and "compares closely with the corresponding bone in other Triassic and Liassic pterosaurs" (pages 126). The ratios of wing phalanx 1/femur and wing phalanx 1/ulna in MFSN 1891 (~1 and ~0.7, respectively [1]), were compared to those of *Preondactylus buffarinii* (considered as 0.90 and 0.73, respectively; actually, they are 1.09 and 0.85 [4]). The slight 'caudal' bending at the distal end of element n was considered to match with those of the wing phalanx 1 in *Peteinosaurus zambellii* (MCSNB 3359) and *Preondactylus buffarinii* (holotype).

The skeletal element as defined by Dalla Vecchia et al. [1] (here see Fig 2A) is an elongate bone that is 27 mm long, with a relatively broad extremity (the supposed proximal, which is about 4 mm wide at maximum) that tapers for 13.5 mm, then maintains the same diameter (1.3 mm) up to the other, broken, extremity (Fig 12B) where it was slightly broader. The bone is straight up to its 'distal' third and then it bends slightly. It is proximally overlapped by the element r. The 'proximal' part is extremely thin, practically a sheet of bone, and shows locally thin, short, and closely spaced longitudinal ridges. It seems to disappear inside a bundle of long bones (r, l1 and an indeterminate element; see Fig 10A). The shaft coming out from beneath the element r is much thicker and is located at a lower level respect to the external surface of the pellet than the purported proximal part. This suggests that the two parts do not belong to a same bone and their continuity is only apparent. This absence of continuity between the two parts is shown also by the 3D rendering of the element. The length of the exposed part of the main portion of the element n (the one that could represent a fragment of a long bone shaft) is 19 mm. This shaft fragment is collapsed in the middle and shows thin longitudinal wrinkles in its broadest part.

The only morphological evidence supporting the identification of this bone as a wing phalanx 1 is its curvature. However, wing phalanx 1 is practically straight in *Preondactylus buffarinii* (see [2]: Figure 3) and does not taper distally like the element n. The left wing phalanx 1 of MCSNB 3359 is bent distally, but it has a comparatively more robust shaft and does not taper distally as the element n; the right wing phalanx 1 of the holotype of *Peteinosaurus zambellii* (MCSNB 2886) is straight. Wing phalanges 1 of MFSN 1797 (*Carniadactylus rosenfeldi*), BSP 1994 I 51 (*Austriadraco dallavecchiai* [50]) and BNM 14524 (*Raeticodactylus filisurensis*) are slightly curved cranially and their shaft is proportionally more robust than that of the bone under examination.

Although not explicitly said by Dalla Vecchia et al. [1], the element n was identified as a wing phalanx 1 because of the outline of its 'proximal' part, which vaguely reminds one of the wing phalanx 1 of early pterosaurs without the process for the extensor tendon. The absence of this process, which is a distinctive trait of wing phalanx 1, could be explained with the immature condition of the individual. In fact, this process is not fused to the phalanx in immature pterodactyloid pterosaurs [51] and could be detached from its original seat. However, this non-fusion was never observed in non-pterodactyloid pterosaurs, also in those showing other evidence of histological immaturity [22, 27, 30–31]. Furthermore, the proximal expanded portion of the wing phalanx 1 is also the thickest of the whole element, while it would be a mere film of bone in the element under examination. The conclusion is that there is no evidence to support the identification of the latter as a pterosaurian wing phalanx 1.

**Wing phalanx 2** (o in Fig 2A)

This element is mentioned only in Figure 3 of Dalla Vecchia et al. [1]. It was probably referred to a wing phalanx because of its straight shaft and the apparent fan-like expansion of one extremity (see [1]: Figure 3). It is a straight segment of a long bone (Fig 11B) whose exposed portion is 13.5 mm long and has a constant diameter (~1.5 mm) up to the left extremity. The latter is partially covered by small bones, and could be fan-shaped (as stated in [1]) or just bent, because its complete outline cannot be observed. The microCT virtual rendering shows just a straight segment. The right extremity is broken.

There is another very similar straight segment of a long bone (labelled x in Fig 2A) close to this segment (Fig 11B), which was not identified in [1]. The exposed portion is 11 mm long and 1.2 m wide; its extremity is evidently broken. As shown by the microCT dataset, the bone does not continue into the pellet, so it is just a shaft fragment.

**Wing phalanx 3** (p in Fig 2A)

This element is also mentioned only in Figure 3 of Dalla Vecchia et al. [1], where the question mark indicates that the identification was considered doubtful. It is a slender, straight bone tapering at the left extremity where it is only 1.1 mm wide (Fig 11C). The other extremity (2.5 mm the maximum width) is blunt and rounded. A thin ridge runs longitudinally in the wider (? proximal) part. The microCT virtual rendering reveals that this element does not continue inside the accumulation, is only 12 mm long, and has a maximum thickness of 0.5 mm in the middle.

The wing phalanx 3 of a Triassic pterosaur would be comparatively more robust and longer compared to the size of the vertebrae of the pellet and, above all, it would have an asymmetrical fan-like proximal part [52–54]. This must be considered just an indeterminate slender bone that is broken at one extremity.

**Wing phalanx 4** (q in Fig 2A)

This element is also mentioned only in Figure 3 of Dalla Vecchia et al. [1], so this identification is not justified by those authors. It was probably based on the expanded and fan-shaped extremity and the relative size respect to the other elements that they referred to wing phalanges. The exposed part of this bone is 6 mm long; the diaphysis is slightly recurved and has a diameter of 1.1 mm (Fig 11D). The other extremity ends against the element r. The segmented 3D rendering shows that the bone does not continue much into the accumulation, so it is probably incomplete.

Theoretically, it could be the proximal part of a wing phalanx 4, but it is most probably the proximal portion of a holocephalous dorsal rib.

**Femur** (r in Fig 2A)

Element r is identified as a femur with doubt in the text of Dalla Vecchia et al. ([1]: pages 125), but it is reported without question mark in Figure 3. The only explanation that those authors give for this identification is that the ratio ulna/femur length would be ~1.38, which is very close to that ratio in *Preondactylus buffarinii* (1.39; actually, it is 1.29 according to [4]).

**Fig 13:**
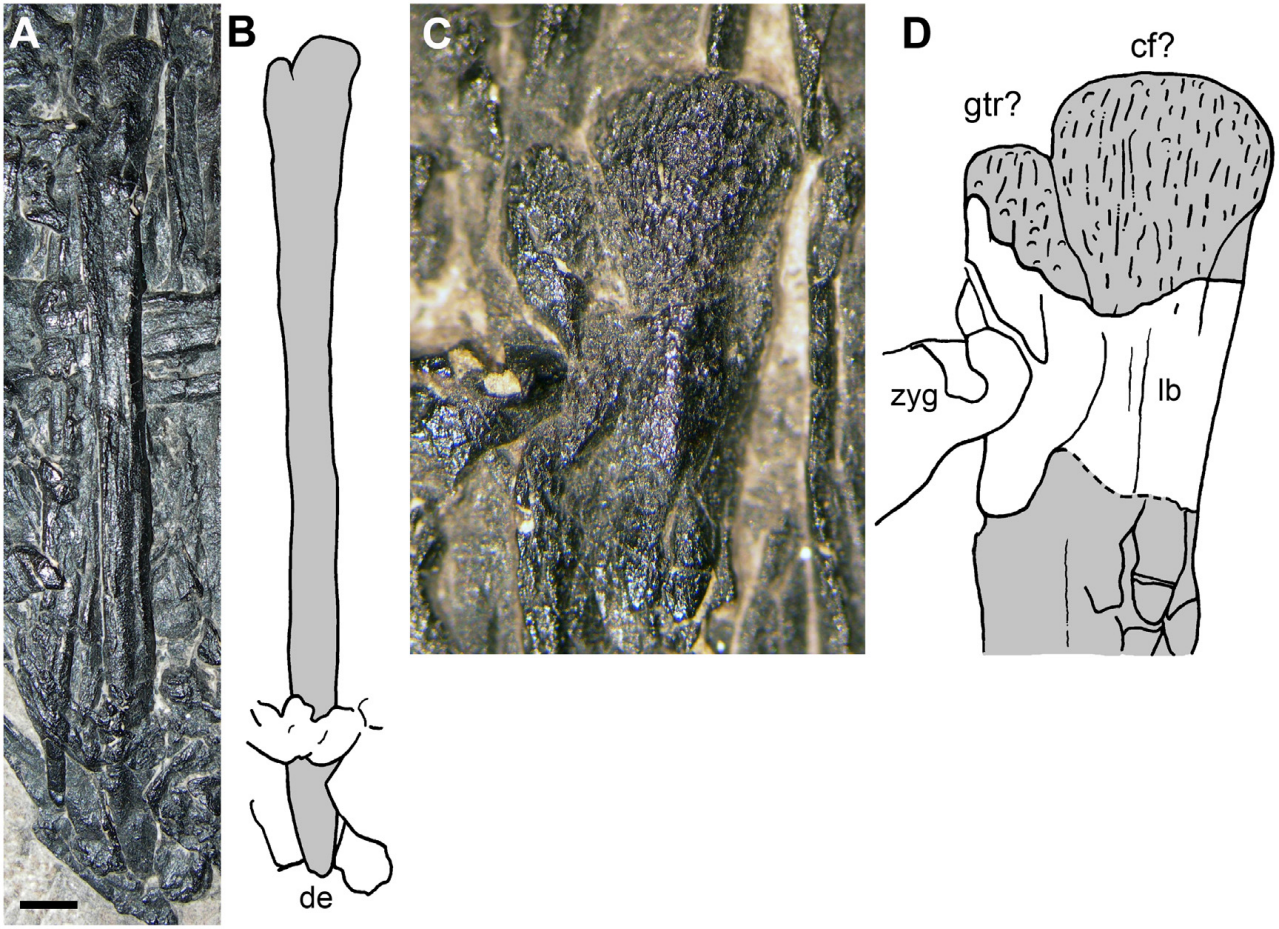
The purported femur (r in Fig 2A). Photograph of the bone (A); interpretative drawing (B); particular of its proximal part (C); interpretative drawing of its proximal part (D). Abbreviations are explained in the text. The scale bar (A) equals 2 mm.

Element r is an elongated and slender bone (Fig 13A and 13B), which is 29 mm long. One exposed extremity (the supposed proximal one, left in Fig 2A) is slightly expanded and appears to be divided into a large hemispherical head and a smaller oval process nearby (Fig 13C and 13D). The surface of both structures is rough and pitted. In the case it is a femur, the larger structure would be the head and the other the greater trochanter. There is no clear evidence of a neck and the head is not angled respect to the shaft. The zone between this extremity and the diaphysis is covered by a very thin sheet of bone and is slightly deformed by the overlapping of a zygapophysis (Fig 13C and 13D). The diaphysis is perfectly straight and tapers slightly in its proximal third up to a diameter of 1.5 mm, then it maintains this diameter up the other extremity (the supposed distal end). The shaft is collapsed in the middle, suggesting that it was hollow. The 'distal' portion is partly covered by other bone fragments; apparently the element tapers toward its 'distal' termination, which is blunt and does not bear evident condyles (Figs 10A, 13A and 13B).

Early pterosaurs have a variable overall femoral shape. The femur of *Eudimorphodon ranzii* (MCSNB 2888) is straight [32], but usually pterosaur femora are slightly sigmoid or recurved [22, 26]. The head of the femur is always angled with respect to the shaft, although in some taxa (e.g., *Eudimorphodon ranzii* and *Carniadactylus rosenfeldi*) the angle is closer to 0 degrees than to 90 degrees [22]. Unfortunately, the distal condyles of the element r are not exposed and cannot be compared with those of the pterosaurian femur.

**Metapodial** (s in Fig 2A)

**Fig 14:**
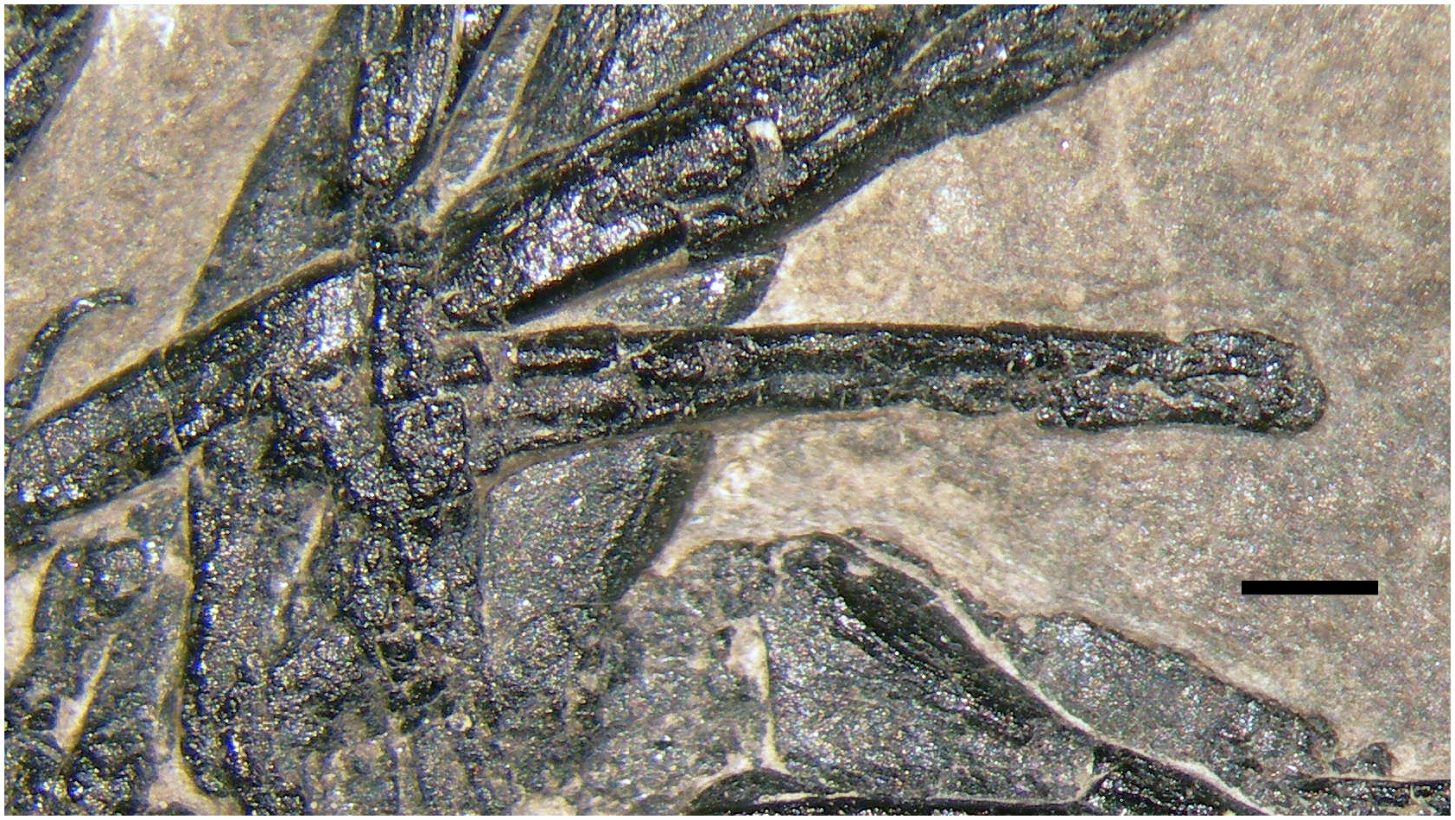
The purported metapodial (s in Fig 2A). Photograph of the bone. The scale bar equals 1 mm.

This element was identified with doubt as a metacarpal or a metatarsal ([1]: Figure 3). It is a slender and slightly recurved bone, which is 6.5 mm long, whose left extremity is covered by some gastralia (Fig 14). The latter is slightly expanded, while the other extremity is not sensibly broader than the shaft and is irregularly blunt.

Usually, the elongate metapodials of pterosaurs, as well as those of the protorosaurians like *Langobardisaurus pandolfii*, are straight and expanded at both extremities [22, 55]. Even if the element s were a broken metapodial, it would not be diagnostic of a pterosaur. However, we suspect that it could be a dorsal rib with the articular head concealed by the gastralia.

## Taphonomical Notes

Dalla Vecchia et al. ([1]: page 126) identified the pellet as the results of the activity of a predator/scavenger based on the breakage of the long bones, which was attributed to its biting action. They identified MFSN 1891 as gastric pellet rather than a coprolite because: 1) bones do not show traces of digestive corrosion, which is typical of coprolites; and 2) bones are not embedded in a fine-grained matrix of organic or phosphatic matter. This aspect can be further developed.

The processes affecting the carcass after the death of a vertebrate are dispersive: it loses pieces up to the complete disarticulation [56–57]. Abiotic processes usually lead to the dispersal of the parts of the body, including the skeletal elements, not to their accumulation. The latter is sometimes caused by high-energy processes (e.g., currents), predator activity (e.g., in caves), mass deaths, or presence of traps, but it concerns the skeletal elements from more individuals in most cases, not the clumping of the disarticulated and partial skeleton of a single, tiny individual [58]. The Dolomia di Forni was deposited in a stagnant and prevailing anoxic intraplatform marine basin at tropical latitudes [20]. The blackish dolostone surrounding the pellet is microlaminated with plane-parallel lamination suggesting deposition in still water without bottom currents and biological reworking. Bioturbation is practically absent in the Dolomia di Forni and there is no evidence of an autochthonous macrofauna [20]. The peculiar bottom conditions allowed the preservation of an allochthonous fossil association composed mainly of poorly mineralized decapod crustaceans, terrestrial plant remains, and fish (often with perfectly articulated skeletons) [20]. Fossils are generally rare and tetrapods are extremely rare [20]. Those conditions could not allow the gathering of the bones from a single individual by currents (because of stagnation) or the casual, abiotic accumulation of the elements from two or more tetrapod individuals (because of stagnation and the extreme rarity of tetrapods).

The clumping of the bones in a mass point to a biotic activity; the preferential orientation of sets of long bones and long bone fragments suggests that the pellet formed in a constrained space as the esophagus or the intestine. The evident breakage of many skeletal elements testifies to the bites of the predator/scavenger before swallowing them. Of course, it is impossible to know whether the swallowed animal was preyed upon or just scavenged. As noted by Dalla Vecchia et al. [1], absence of gastric acid corrosion and of a background of a fine-grained organic or phosphatic matter (as the case of the abundant fish coprolites found in the Dolomia di Forni; [20]) suggest that MFSN 1891 is a regurgitation not a fecal pellet.

As for the possible predator/scavenger, the present state of knowledge of the Dolomia di Forni fossil assemblage [20] suggests it could be a large fish, perhaps *Saurichthys* or a coelacanthiform (both found in the same section as MFSN 1891), or the rarer *Birgeria*. No records about the production of gastric pellets consisting of vertebrate remains are reported for living fishes, whereas they exist for birds, crocodiles and carnivorous mammals [59]. However, they are tentatively reported from the fossil record. A gastric pellet (Speiballen in German) with pterosaur bones from the Upper Jurassic of Germany was referred as a prey of a large fish or a marine crocodile [7]. Remains of actinopterygian fish from the Upper Jurassic of Germany were also interpreted as gastric ejecta produced by a large fish or a crocodile [60–61]. The producer of a gastric pellet containing fish remains from the Lower Jurassic of Germany was tentatively referred to a fish or marine reptile [59]. Contrary to what suggested by Myhrvold [11], the pellet could not have been produced by a pterosaur, a marine reptile or a crocodilian because: 1) Triassic pterosaurs were not large enough to swallow an animal the size of that represented by the bones preserved in MFSN 1891; 2) no marine predatory reptiles, such as eusauropterygians or ichthyosaurians, have ever been found in the Dolomia di Forni and coeval units of a wide area around the outcrop of the Dolomia di Forni basin; and 3) there is no evidence of the presence of the first primitive crocodylomorphs in the carbonate platform surrounding the basin [20] and Triassic crocodylomorphs were relatively small and unlike the living crocodilians [62].

## Discussion

The analysis bone by bone does not find support for the hypothesis that the skeletal elements in the pellet belong to a pterosaur. The identification of the presumed caudal vertebra as an elongate cervical and the Y-like hemapophysis would suggest that the bones in the pellet are not those of a pterosaur.

If not a pterosaur, whose are the bones in the pellet? The candidate is a small-sized reptile (neural arches are fused, although possibly with nearly obliterated suture, and skeletal elements are well ossified, so it is not an hatchling or an early juvenile), with rather elongate, probably procoelous cervical vertebrae with low neural arch and spine; filiform cervical ribs; procoelous dorsal vertebrae; at least some dicephalous dorsal ribs; Y-like chevrons; gastralia; elongated and hollow limb bones; and probably no osteoderms. Small size and absence of osteoderms exclude groups as phytosaurians, aetosaurians, bizarre basal archosauriforms as doswellids and *Vancleavea*, and possibly most pseudosuchians. These groups are excluded also by the absence of procoelous dorsals and elongated cervicals. Actually, very few of the Middle-Late Triassic reptiles that we take as reference for comparison are reported to have procoelous dorsals in the literature: just pterosaurs and some protorosaurians (*Langobardisaurus* [47], *Tanytrachelos* [63]). The presence of procoelous vertebrae in Triassic microvertebrate assemblages [64–65] could suggest that other, unknown reptiles had procoelous dorsals. Anyway, we restrict our comparison to named taxa. It is also true that the condition of the dorsal vertebral articulation is unknown in some of the listed taxa (e.g., *Mecistotrachelos*, *Sharovipteryx*, and *Longisquama*). However, the gliding diapsid *Mecistotrachelos* could be excluded because its dorsals are characteristically elongate and it would preserve a record of its extremely elongated ribs [66].

Some drepanosauromorphs (*Drepanosaurus*, *Hypuronector* and possibly also *Dolabrosaurus*) do not have procoelous dorsals. The condition is not clear in *Megalancosaurus* (which was also found in the Dolomia di Forni; [67]) and *Vallesaurus*. The articular surface of the centrum in the only dorsal vertebra of *Megalancosaurus* where this surface can be observed seems to be convex but with a deep depression in the middle (S. Renesto, pers. comm. May 2015); this caudal articular portion would not appear unlike that of the elements f1 and f2 in lateral view. However, the dorsal ribs and the hemapophyses are fused to the vertebrae in drepanosauromorphs ([68]; S. Renesto, pers. comm.) and the hemapophyses often have peculiar morphologies (*Drepanosaurus*, *Megalancosaurus*, *Dolabrosaurus*, and *Hypuronector*). Furthermore, cervicals of drepanosauromorphs have large hypapophyses projecting from the caudal part of the centrum and relatively narrow neural spines that are inclined cranially [68]. So, drepanosauromorphs can be excluded as candidates for the pellet.

*Langobardisaurus* (which was also found in the Dolomia di Forni; [69]) and *Tanytrachelos* have elongated and procoelous cervical vertebrae with low neural arch (Fig 7F) as those of MCSNB 1891 [47, 63]. Also the cervical and caudal vertebrae of *Sharovipteryx* are elongated [70], while those of *Longisquama* are short [71]. It is unknown whether the cervicals of *Sharovipteryx* are procoelous; however, they bear long filiform ribs. Furthermore, *Sharovipteryx* has very elongated and probably hollow hind limb bones [70]. Unfortunately, too few information is available on *Sharovipteryx* for further comparison.

The low and rectangular neural spine of the element c is reminiscent of the cervical vertebrae of some protorosaurians (*Macrocnemus* spp., [72–73], *Langobardisaurus pandolfii* [47]). However, the laterally projecting process is something unique, lacking in the cervical vertebrae of the protorosaurians. The dorsal vertebrae of the protorosaurians have narrow transverse processes, but they are shorter and their neural spines are taller.

At least some protorosaurians (*Langobardisaurus*, [74]; *Tanystropheus*, FMDV pers. obs.) have hollow limb bones. The limb bones of *Langobardisaurus* and *Macrocnemus* are comparatively slender and elongated, mainly the femur, tibia, and fibula. Some protorosaurians have proximal dicephalous and distal holocephalous dorsal ribs (*Langobardisaurus* [47]; *Tanytrachelos* [63]; and *Tanystropheus* [33]).

Thus, morphological comparison suggests a closer resemblance to protorosaurians.

As for size, the vertebrae of the pellet are in between those of the larger and presumably adult specimens of *Langobardisaurus pandolfii* (MCSNB 2883 and MFSN 1921) and those of the small Austrian specimen (P 10121; Table 1) [74]. The cervical vertebra (h1) appears to be comparatively shorter with respect to the dorsal vertebra than in *Langobardisaurus pandolfii* specimens.

**Table 1:** Measurements (in mm) of skeletal elements in MFSN 1891 and in *Langobardisaurus* specimens.

elements	MFSN 1891	MCSNB 2883	MFSN 1921	MFSN 26829	MCSNB4860	P 10121
Cervical vertebrae	12.4/**11**	**16.5/19** (6-7th)	~16 (6th)	-	-	**6.3** (6th)
Dorsal vertebrae	7/**5.1**	**7.5** (M)	8–6?	-	-	-
Humerus	-	-	36.5	-	18	19.1
Radius	-	-	27	-	12	12.3
Ulna	-	-	26.5	-	12.5	14.9
Femur	-	57(M)	49	54	27	28.9
Tibia	-	47	45.5	42	24	26.3
Fibula	-	44	42.5	42	23	26.3
Metatarsal III	-	-	22	22	10.5	10.4
k1	~39.5					
k2	~29					
l1	>36					
l2	23					
R	29					
N	>19					
O	>13.5					
X	>10.3					
P	>12					
hsf1	>19					
hsf2	>18					
hsf3	>16					

Measurements based on Renesto [47] and Saller et al. [74] and direct observation of the specimens. The vertebral lengths based on centrum length only (according to S. Renesto pers. comm. in *Langobardisaurus*) are reported in bold type. Abbreviations: M, mean.

Dalla Vecchia [18] considered the referral to a pterosaur as the most parsimonious hypothesis because of the presence of many long elements of the limbs. At least 12 segments of long bones have been identified in MFSN 1891 (k1-2, l1-2, n, o, p, r, x, and the three hsf1-3 in the hidden side), possibly 14 counting also those grouped around the element l1. As there are usually 12 long limb bones in a tetrapod (humerus, radius and ulna in the forelimb, femur, tibia and fibula in the hind limb), which can match the elongation of the fragments found in the pellet, this number is high, but not necessarily supporting a pterosaur identification (a pterosaur has 20 long limb bones because of the eight very elongated wing phalanges).

**Fig 15:**
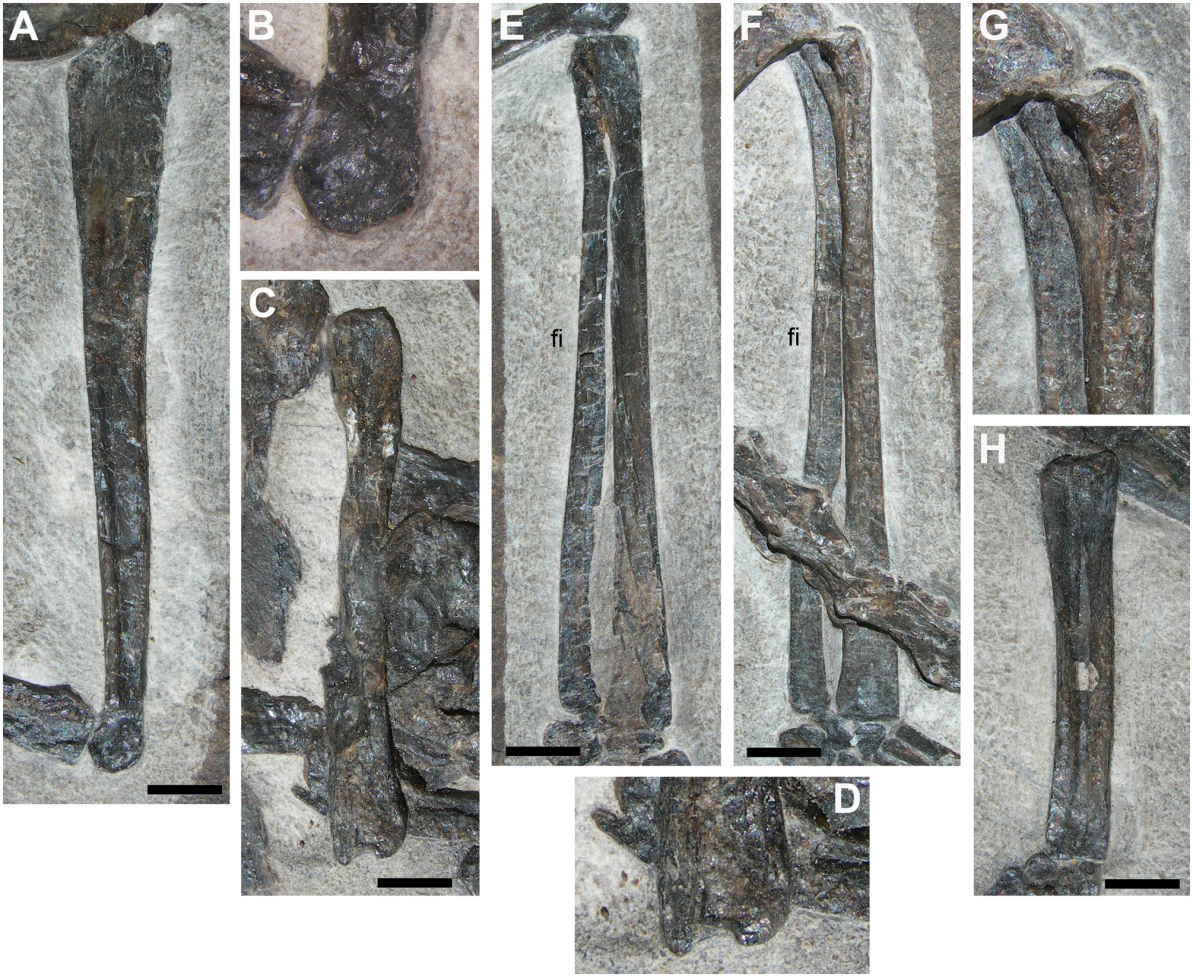
Limb bones of the protorosaurian *Langobardisaurus pandolfii* (MFSN 1921). The right femur (A); particular of the distal articular condyle of the right femur (B); the right humerus (C); the distal condyles of the right humerus (D); right tibia and fibula (E); left tibia and fibula (F); particular of the proximal portion of the left tibia (G); right radius and ulna (H). Abbreviations are explained in the text. The scale bar equals 5 mm.

The longest limb element in *Langobardisaurus pandolfii* is the femur, which is 3–3.45 times the length of an elongate cervical vertebra and 7.6 times the length of a dorsal vertebra (see Table 2) [47]. The longest long bone in the pellet is apparently k1, which is 3.19/3.59 times the length of h1 (the higher value was obtained considering the centrum length as proxy for vertebral length as done by Renesto [47]; S. Renesto, pers. comm. May 2015) and 7.9 times the length of the dorsal vertebra (f1) considering only the centrum as proxy for vertebral length (Table 2). The overall morphology of k1, as well as the shape of its distal condyle and the slight distal curvature of the shaft resembles those observed in the femur of *Langobardisaurus pandolfii* (Fig 15A and 15B). So, k1 is morphologically and proportionally similar to a femur of *Langobardisaurus pandolfii*. The element f1 is the second longest bone in the pellet (36 mm, but broken at both extremities), so it could be the other femur. If the element r is a femur, it is unlike that of *Langobardisaurus pandolfii*, which lacks a distinct head and a prominent greater trochanter (Fig 15A) [47, 55]. However, the apparent separation of head and greater trochanter in MFSN 1891 could be just the results of the breakage of a hemispherical proximal articular head. As for proportions with the vertebrae, r could be a *Langobardisaurus* humerus (Table 2), which is also a straight element (Fig 15C). On the same base, k2 could be a tibia (Fig 15E and 15F); l2 could be a radius or ulna, but its apparently bicondylar end would match with the morphology of the distal end humerus (Fig 15D) or, to a lesser extent, to the proximal end of the tibia (Fig 15G). The curvature of the element n is apparently contrasting with the straight limb bones of *Langobardisaurus* (see [46]). However, the fibula of MFSN 1921 shows a slight distal curvature (Fig 15E and 15F), so the element n can be tentatively referred to a fibula. The other bones of the *Langobardisaurus* skeleton (a tibia, a humerus, a fibula, a radius, an ulna, and a radius or an ulna; see Fig 15H) could be well represented by the six fragments o, p, x and hsf1-3. This confirms that the number of long bone fragments is compatible with a protorosaurian, although the morphological support that it is a *Langobardisaurus pandolfii* is not strong.

**Table 2:** Ratios between long bone lengths and cervical and dorsal vertebrae length in MFSN 1891.

ratio	MFSN 1891	MCSNB 2883	MFSN 1921	P 10121
fe/cv		**3.00/3.45**	3.06	**4.58**
fe/dv		**7.60**	8.0/6.15	-
h/cv	-	**-**	2.26	**3.03**
h/dv	-	**-**	6.08/4.56	-
ra/cv	-	**-**	1.69	**1.95**
ra/dv	-	**-**	4.50/3.37	-
u/cv	-	**-**	1.66	**2.35**
u/dv	-	**-**	4.42/3.30	-
ti/cv	-	**2.85/2.47**	2.84	**4.17**
ti/dv	-	**6.25**	7.58/5.69	-
fi/cv	-	**2.66/2.30**	2.66	**4.17**
fi/dv	-	**5.87**	7.08/5.31	-
k1/cv	3.19/**3.59**			
k1/dv	5.64/**7.6**			
l1/cv	>2.90; >**3.27**			
l1/dv	>5.14; >**7.06**			
r/cv	2.34/**2.64**			
r/dv	4.14/**5.69**			
k2/cv	>2.34; >**2.64**			
k2/dv	>4.14;>**5.69**			
n/cv	>1.53;>**1.73**			
n/dv	>2.71;>**3.72**			
o/cv	>1.09;>**1.23**			
o/dv	>1.93;>**2.65**			
x/cv	>0.83;>**0.94**			
x/dv	>1.47;>**2.02**			
l2/cv	1.85/**2.09**			
l2/dv	**4.51**/3.29			
hsf1/cv	>1.53;>**1.73**			
hsf2/cv	>1.45;>**1.64**			
hsf3/cv	>1.29;>**1.45**			
hsf1/dv	>2.71;>**3.73**			
hsf2/dv	>2.55;>**3.53**			
hsf3/dv	>2.29;>**3.14**			

The ratios obtained from vertebral lengths based on centrum length only are reported in bold type.

The presence of a protorosaurian reptile, which presumably was terrestrial, in a marine formation is not unexpected. The fossil record from the Dolomia di Forni Formation includes abundant remains of terrestrial organisms as well as organic matter of terrestrial origin [20]. The frequent plant leaves, small branches and cones (mainly belonging to conifers [20]), the spider *Friularachne rigoi* Dalla Vecchia and Selden 2013 [75], the drepanosauromorph *Megalancosaurus preonensis*, the protorosaurian *Langobardisaurus pandolfii* and a probable lepidosauromorph [76] were all allochthonous. They lived on the emergent parts of the carbonate platform surrounding the anoxic basin of the Dolomia di Forni Formation and were transported into it by tidal currents, storms or hurricanes, or—the animals—on floating plant remains [75].

## Conclusions

The microCT acquisition allowed to properly observe the surface of the pellet preserved inside the rock, but its utility in distinguishing the packed skeletal elements was limited. The best candidate for the pellet is not a pterosaur, but a protorosaurian like *Langobardisaurus*. However, the morphology of the described elements does not match perfectly with those of *Langobardisaurus pandolfii* found in the same Dolomia di Forni Formation. Therefore, the skeletal remains of MFSN 1891 could belong to a similar, but distinct, protorosaurian taxon, or to a still unknown small reptile with procoelous dorsal vertebrae, rather elongate and probably procoelous cervical vertebrae with low neural arch and spine, filiform cervical ribs, at least some dicephalous dorsal ribs, elongated and hollow limb bones, and no osteoderms.
